# Contaminants of emerging concern in tributaries to the Laurentian Great Lakes: I. Patterns of occurrence

**DOI:** 10.1371/journal.pone.0182868

**Published:** 2017-09-27

**Authors:** Sarah M. Elliott, Mark E. Brigham, Kathy E. Lee, Jo A. Banda, Steven J. Choy, Daniel J. Gefell, Thomas A. Minarik, Jeremy N. Moore, Zachary G. Jorgenson

**Affiliations:** 1 U.S. Geological Survey, Mounds View, Minnesota, United States of America; 2 U.S. Geological Survey, Grand Rapids, Minnesota, United States of America; 3 U.S. Fish and Wildlife Service, Columbus, Ohio, United States of America; 4 U.S. Fish and Wildlife Service, Madison, Wisconsin, United States of America; 5 U.S. Fish and Wildlife Service, Cortland, New York, United States of America; 6 Metropolitan Water Reclamation District of Greater Chicago, Cicero, IL, United States of America; 7 U.S. Fish and Wildlife Service, Chubbuck, Idaho, United States of America; 8 Department of Biology, St. Cloud State University, St. Cloud, Minnesota, United States of America; 9 U.S. Fish and Wildlife Service, Bloomington, Minnesota, United States of America; Purdue University, UNITED STATES

## Abstract

Human activities introduce a variety of chemicals to the Laurentian Great Lakes including pesticides, pharmaceuticals, flame retardants, plasticizers, and solvents (collectively referred to as contaminants of emerging concern or CECs) potentially threatening the vitality of these valuable ecosystems. We conducted a basin-wide study to identify the presence of CECs and other chemicals of interest in 12 U.S. tributaries to the Laurentian Great Lakes during 2013 and 2014. A total of 292 surface-water and 80 sediment samples were collected and analyzed for approximately 200 chemicals. A total of 32 and 28 chemicals were detected in at least 30% of water and sediment samples, respectively. Concentrations ranged from 0.0284 (indole) to 72.2 (cholesterol) μg/L in water and 1.75 (diphenhydramine) to 20,800 μg/kg (fluoranthene) in sediment. Cluster analyses revealed chemicals that frequently co-occurred such as pharmaceuticals and flame retardants at sites receiving similar inputs such as wastewater treatment plant effluent. Comparison of environmental concentrations to water and sediment-quality benchmarks revealed that polycyclic aromatic hydrocarbon concentrations often exceeded benchmarks in both water and sediment. Additionally, bis(2-ethylhexyl) phthalate and dichlorvos concentrations exceeded water-quality benchmarks in several rivers. Results from this study can be used to understand organism exposure, prioritize river basins for future management efforts, and guide detailed assessments of factors influencing transport and fate of CECs in the Great Lakes Basin.

## Introduction

Human activities introduce complex mixtures of organic contaminants into natural waters. Risks associated with persistent contaminants such as dichlorodiphenythrichloroethane (DDT), polychlorinated biphenyls (PCBs), and many other organochlorines have been recognized since the 1960s, and regulatory action has been effective at greatly reducing the occurrence of many of these persistent organic pollutants in the environment. More recently, however, the scientific community has recognized that many additional classes of organic chemicals are ubiquitous in natural waters, including current-use pesticides, pharmaceuticals, flame retardants, and solvents [1,2]. With increasing knowledge about the ubiquitous presence of CECs in the environment, attention is now being focused on valuable freshwater resources such as the Laurentian Great Lakes (hereafter referred to as ‘Great Lakes’).

The Great Lakes Basin supported a population of over 30 million people in 2015 [3]. Major industries have developed in some of the Basin’s larger population centers such as Chicago, Illinois; Detroit, Michigan; and Cleveland, Ohio. Additionally, row-crop and livestock agriculture are prevalent land uses within the Basin. Even though many of the surface waters within the Basin receive stormwater, wastewater, and agricultural runoff, the waters are still relied upon for public use, including drinking sources. In 2015, approximately 12 billion L/day were withdrawn from the U.S. portion of the Great Lakes basin for public water use [3]. The Great Lakes also support an important recreational, subsistence, and commercial fishery. Because of the reliance of the U.S. on the Great Lakes Basin, threats to this resource are of considerable importance.

Contaminants of emerging concern have been identified in several Great Lakes. For example, pharmaceuticals and personal care products were detected at concentrations of potential concern to aquatic organisms in Lake Michigan up to 3.2 km away from a major WWTP outfall [4]. Polybrominated flame retardants have been detected in all five Great Lakes, with increasing loading rates in recent years [5–9]. In addition to the identification of CECs in the Great Lakes themselves, several CECs and other chemicals of interest have been detected in U.S. tributaries to the Great Lakes, including detergent metabolites, pesticides, and polycyclic aromatic hydrocarbons (PAHs) [10,11]; many of which were detected at concentrations that potentially pose a risk to aquatic biota. Potential risks include intersex, increased stress, and behavioral changes. Although these risks are not well defined, they could have important implications for sustaining fish and wildlife populations.

In addition to the recreational and subsistence values of the Great Lakes, the Basin provides habitat for a diversity of wildlife, some threatened or endangered. Exposure to CECs has been evident in various biota including bald eagle nestlings [12–14], several fish species [15–17], and several mussel species [18–20]. Although there is evidence that organisms are being exposed to and can bioaccumulate CECs, there is limited information relating exposure to effects in the wild. This is especially true for listed (e.g. threatened or endangered), commercial, or sport wildlife important to the Great Lakes community. Understanding how these contaminants may be affecting fish and wildlife is important for the management of these important resources.

As part of a long-term project, water and sediment samples were collected from U.S. tributaries to the Great Lakes to: (1) assess the occurrence and magnitude of a broad suite of CECs and other chemicals of interest and (2) better understand aquatic organism exposure. This paper reports occurrence and concentration data for CECs and other chemicals of interest collected throughout the U.S. portion of the Great Lakes Basin during 2013–14. Patterns of chemical occurrence were explored using a combination of univariate and multivariate statistics, as well as basic landscape-scale variables such as land use and presence of point sources. Data were also used to calculate estimates of estrogenicity and compared with benchmarks or known effects, when available, to highlight potential risks to aquatic biota. A companion paper [21] reports relationships between CEC occurrence and biological data to better understand the effects of CECs on aquatic biota.

## Materials and methods

### Study area

The Great Lakes—Superior, Michigan, Huron, Erie, and Ontario—and St. Lawrence River represent the largest surface freshwater supply in the world [3]. Collectively, the Lakes drain an area of 750,000 km^2^ in the northeastern United States and eastern Canada. Predominant land cover varies within the Basin and includes urban, agricultural, and forest. Sample sites were chosen to represent a mix of urban and agricultural land uses, while a subset of study sites represent forest and wetland dominated watersheds with relatively little human disturbance. Sites located in predominantly urban areas are affected by overland runoff from the urban landscape and direct discharge of storm drains and combined sewer overflows (CSOs), as well as treated wastewater effluent. Sites located in predominantly agricultural areas are affected by row crop production and/or animal feeding operations.

### Sampling methods

**Fig 1:**
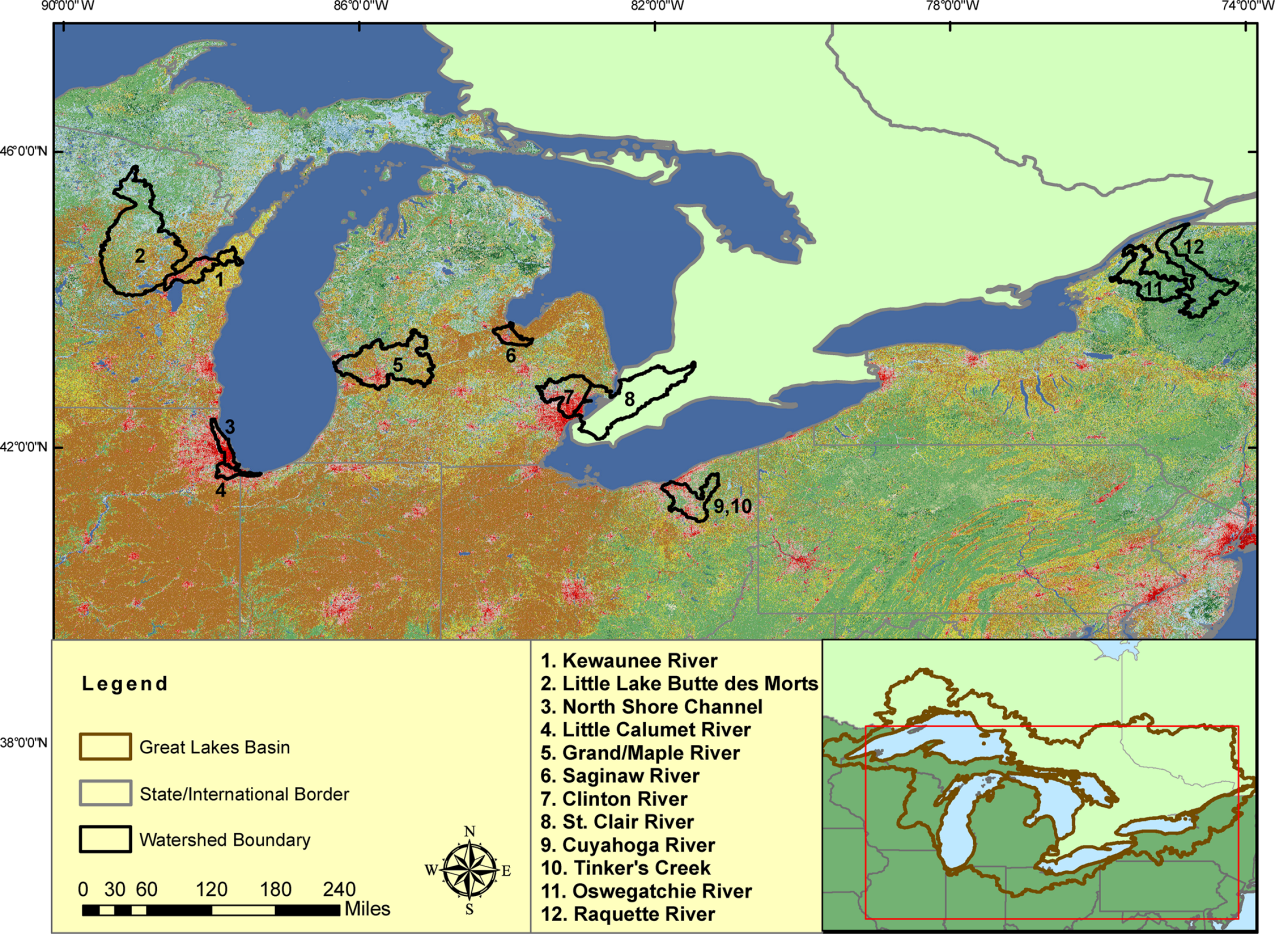
Great Lakes Basin map showing U.S. tributaries sampled in 2013–14. Numbers indicate the river basin sampled within the designated watershed. Colors depict land use as described in Homer et al. [26]. Generally, red/pink represent developed, yellow/brown represent agriculture, greens are forest, and blues are wetlands and open water.

A total of 292 surface-water and 80 bottom-sediment samples were collected from 12 U.S. tributaries to the Great Lakes (Fig 1) during 2013 and 2014. A list of study sites and samples collected is provided in S1 Table. Permissions for access to field sites were secured by the U.S. Fish and Wildlife Service. Maps of specific study site locations are provided in Figs A-K in S2 File. In general, sites were sampled twice: once in spring to coincide with fish spawn and once in late summer to coincide with low flow. Sampling methods followed those detailed by Lee et al. [22,23] and Elliott et al. [24,25]. Briefly, water samples were collected from streams and lakes using a modified depth-integrated sampling method with a weighted-bottle sampler fitted with a 1-L, baked amber glass bottle. Bottom-sediment samples were collected to include most recent deposition (approximately top 10 cm) using a stainless steel Ekman dredge. All samples were collected with inert materials, and sampling personnel avoided the use of topical personal-care items such as insect repellent, cologne, aftershave, and perfume. Samples were stored at about 2°C until they were shipped overnight on ice to the analyzing laboratory.

### Analytical methods

Water samples were analyzed at the U.S. Geological Survey National Water Quality Laboratory (NWQL) for 69 wastewater indicator chemicals, 20 steroid hormones and sterols, and 110 pharmaceuticals. Wastewater indicator chemicals in unfiltered water were determined by continuous liquid–liquid extraction and capillary-column gas chromatography/mass spectrometry (GC/MS) following the method of Zaugg et al. [27]. Steroid hormones, sterols, and bisphenol A in unfiltered water were determined by solid-phase extraction, derivatization, and gas chromatography with tandem mass spectrometry (GC/TMS) following the method of Foreman et al. [28]. Pharmaceuticals in filtered water were analyzed by direct aqueous injection high-performance liquid chromatography/tandem mass spectrometry as described in Furlong et al. [29].

Bottom-sediment samples were analyzed for 57 wastewater indicator chemicals, 20 steroid hormones and sterols, and 31 pharmaceuticals at the NWQL. Wastewater indicator chemicals were extracted by high-pressure water/isopropyl alcohol extraction. Chemicals were isolated with solid-phase extraction and determined by capillary-column GC/MS as described by Burkhardt et al. [30]. This method was adapted for determination of steroid hormones, sterols, and bisphenol A in bottom sediment as described by Fischer et al. [31]. Pharmaceuticals and antidepressants were extracted by accelerated solvent extraction techniques and determined by high performance liquid chromatography according to a method developed for biosolids [32,33], which was adapted for sediment samples as described in Lee et al. [23].

All data for this study, further details on field and analytical methods, and a summary of quality-control data are available online [24,25].

### Data analysis

Data reduction and summary statistics were completed using SAS® software, version 9.3 [34]. Data reporting conventions for the methods used in this study are detailed in Childress et al. [35]. Briefly, a long-term method detection limit (LT-MDL) is defined for each chemical as the standard deviation of at least 24 analyses of low-level standards per year, across multiple instruments, and multiple analysts. A laboratory reporting limit (LRL) is set at 2•LT-MDL. Analytical determinations (results) ≥ LRL have a <1% chance of being a false positive; there is also a ≤1% chance of a false negative. Results ≥ LRL are generally reported without qualifiers. Quantifiable detections < LRL are reported as estimated (an ‘E’ accompanies the numerical result). For the concentration range LT-MDL< result < LRL, there is still ≤1% chance of false positives, although there is an increasing probability of false negatives as concentrations decrease within this range (at the LT-MDL there is a 50% chance of a false negative). The laboratory analytical methods used for this study are characterized as “information rich” by NWQL, meaning confirmatory evidence that a given chemical is present (such as a mass spectrum that is diagnostic of a specific chemical) may yield an estimated result that is less than the LT-MDL [35]. There is greater uncertainty in results that are less than the LT-MDL. Although such data often reveal interpretable patterns, they should not be the foundation of regulatory actions. For purposes of data reduction, left-censored results were re-coded as zeroes. This recoding was deemed appropriate for rank-transformation and other summary statistics used in this analysis.

Data were reduced to one observation per site, representing the maximum concentration of each chemical detected at each site. Maxima were used for several reasons. First, as a screening exercise, maximum concentrations are more likely to capture conditions of greatest toxicological concern. Second, because there was an abundance of left-censored data and each site was sampled only a few times, data were generally insufficient for calculating mean concentrations using techniques such as maximum likelihood estimation (which require distributional assumptions of the data). Next, chemicals were categorized by chemical class (e.g. pharmaceutical, pesticides, etc.) and the concentrations of all chemicals detected within a given class at each site were summed to provide total chemical class and total site concentrations. Because total chemical class and site concentrations are based on the maximum concentration of each chemical detected in multiple samples, these calculations may be biased high. Chemicals that did not fit into a natural class (e.g. pharmaceutical, pesticide, etc.) were placed into an ‘other’ class. Lastly, detection frequency, as well as, median and maximum concentrations were calculated for each chemical. The dataset was further reduced to only include those CECs that were detected in at least 30% of samples across the Great Lakes Basin. The 30% detection frequency threshold was chosen so that the dataset included CECs that are fairly ubiquitous across the Basin. Although this reduction method may eliminate information regarding CEC-specific occurrences for those present in <30% of samples, it was deemed appropriate for this broad-scale survey.

Because many CECs exhibit estrogenic activity, estradiol equivalents (EEQ) were calculated for water and sediment at each site to estimate potential estrogenicity. These calculations provide an indication of the cumulative effects of estrogenic chemicals that may result in endocrine-disrupting effects. The EEQ was calculated based on the maximum concentration observed for each chemical that had a literature EEQ factor. Factors representing a chemical’s potency relative to 17β-estradiol were multiplied by the maximum concentration of each applicable chemical. A total EEQ was obtained for each site by summing the individual EEQs of each chemical measured at each site. The EEQ factors for individual chemicals are presented in the companion to this paper [21].

Based on maximum concentrations of individual chemicals per site and a detection frequency of at least 30%, cluster analyses were used to assess patterns of chemical occurrence. Clusters were generated using hierarchical clustering of Euclidian distance matrices on rank-transformed data. Two different cluster analyses were completed to identify: (1) CECs that were often detected together and (2) sites with similar CEC signatures. Heatmaps with cluster analysis dendrograms were generated in R software (version 3.1.3) [36] using the heatmap.2 function in the gplots package [37]; further details are in Supporting Information. Only CECs detected in ≥30% (Table 1) of the samples were included in cluster analyses to represent those that are fairly ubiquitous throughout the Basin.

**Table 1:** Chemicals of emerging concern detected in at least 30% of all surface-water and sediment samples collected from U.S. tributaries to the Great Lakes, 2013–14.

Chemical		Chemical class	K_ow_^[^^46^^]^	Reporting Limit	N	Median concentration	Maximum concentration	Detection frequency, percent
	*Water (micrograms per liter)*							
Cholesterol		Sterol	7.69	0.20	285	1.80	72.2	100
Isophorone		Other	1.7	0.05	291	0.0068	0.0548	72
Metformin		Pharmaceutical	-0.88	0.013	288	0.0465	33.6	71
Atrazine		Pesticide	2.61	0.019	292	0.0184	0.814	70
DEET		Insect Repellant	2.18	0.04	291	0.021	5.07	68
Metolachlor		Pesticide	3.13	0.04	291	0.0136	1.53	62
β-Sitosterol		Sterol	8.25	4.8	291	0.42	7.77	61
Carbamazepine		Pharmaceutical	2.45	0.0041	292	0.00128	0.333	52
TBEP		Flame retardant	2.75	0.64	291	0.105	38.7	52
Lidocaine		Pharmaceutical	1.44	0.015	292	0.00015	2.06	50
BTZA		Industrial	1.44	0.141	292	0.004	8.06	50
Desvenlafaxine		Pharmaceutical	--	0.00749	292	<RL	1.24	48
Methocarbamol		Pharmaceutical	0.61	0.00872	292	<RL	0.592	47
Tramadol		Pharmaceutical	2.38	0.0151	292	<RL	0.863	46
Cotinine		Other	0.07	0.00637	292	<RL	0.121	45
HHCB		Fragrance	5.9	0.04	291	<RL	2.18	44
Indole		Fecal indicator	2.14	0.04	289	<RL	0.0284	44
Fexofenadine		Pharmaceutical	3.88	0.0199	292	<RL	3.62	43
9,10-Anthraquinone		Industrial	3.39	0.04	291	<RL	0.402	42
Venlafaxine		Pharmaceutical	2.51	0.00448	292	<RL	0.319	42
Caffeine		Other	-0.07	0.0907	292	<RL	6.61	40
TDIP		Flame retardant	3.65	0.32	291	<RL	0.44	37
Nicotine		Other	1.17	0.0578	292	<RL	0.492	37
Acyclovir		Pharmaceutical	-1.56	0.0222	292	<RL	1.11	36
Fluoranthene		PAH	5.16	0.02	291	<RL	0.671	36
Sulfamethoxazole		Pharmaceutical	0.89	0.0261	292	<RL	1.39	36
Triamterene		Pharmaceutical	0.98	0.00525	292	<RL	0.382	36
Pyrene		PAH	4.88	0.02	291	<RL	0.512	35
Atenolol		Pharmaceutical	0.16	0.0133	292	<RL	1.70	34
3β-Coprostanol		Sterol	8.22	1.6	291	<RL	14.4	34
Meprobamate		Pharmaceutical	0.7	0.086	292	<RL	0.114	32
Metoprolol		Pharmaceutical	1.88	0.0275	292	<RL	0.415	30
	*Sediment (micrograms per kilogram)*							
Indole		Fecal indicator	2.14	50	76	130	1,240	100
3-Methyl-1*H*-indole		Fecal indicator	2.6	33	76	10.7	574	91
Fluoranthene		PAH	5.16	41.5	76	192	20,800	87
Pyrene		PAH	4.88	41.5	76	165	18,300	87
Benzo[a]pyrene		PAH	6.13	41.5	76	65.3	1,790	86
2,6-Dimethylnaphthalene		PAH	4.31	33	76	25.5	338	84
Carbazole		Other	3.72	34.5	76	15.4	678	80
Anthracene		PAH	4.45	40	76	15.5	918	76
Phenanthrene		PAH	4.46	37.5	76	112.5	2,840	74
9,10-Anthraquinone		Industrial	3.39	33	71	46.1	899	73
*p*-Cresol		Phenolic	1.94	165	76	73.2	9,330	72
Estrone		Hormone	3.13	0.053	76	0.32	10.93	68
A4		Hormone	2.75	0.053	76	0.171	2.85	67
Cholesterol		Sterol	7.69	165	76	586	5,400	54
1-Methylnaphthalene		PAH	3.87	33	76	1.045	197	50
2-Methylnaphthalene		PAH	3.86	33	76	<RL	406	49
HHCB		Fragrance	5.9	33	76	<RL	660	46
β-Sitosterol		Sterol	8.25	330	76	<RL	15,200	43
Naphthalene		PAH	3.3	33	76	<RL	1,720	43
β-Stigmastanol		Sterol	8.63	330	76	<RL	5,250	42
Isophorone		Other	1.7	33	76	<RL	46.2	41
4-*tert*-Octylphenol		Alkylphenol	5.28	33	75	<RL	169	37
Bisphenol A		Plasticizer	3.32	33	76	<RL	380	37
17β-Estradiol		Hormone	4.01	0.053	74	<RL	5.85	36
*cis*-Androsterone		Hormone	3.69	0.053	76	<RL	6.91	36
Diphenhydramine		Pharmaceutical	3.27	2.7	77	<RL	1.75	32
1,4-Dichlorobenzene		Other	3.44	33	76	<RL	1,160	30
AHTN		Fragrance	5.7	33	76	<RL	104	30

N, number of samples; DEET, *N*,*N-*Diethyl-m-toluamide; TBEP, tris(2-butoxyethyl) phosphate; BTZA, methyl-1*H*-benzotriazole; --, no data; <RL, less than reporting level; PAH, polycyclic aromatic hydrocarbon; HHCB, hexahydrohexamethyl cyclopentabenzopyran; TDIP, tris(dichloroisopropyl) phosphate; A4, 4-androstene-3,17-dione; AHTN, acetyl hexamethyl tetrahydronaphthalene.

### Comparisons to benchmarks

Comparisons of the environmental data to established benchmarks can provide context for CEC concentrations in relation to expected adverse effects to aquatic biota. Agencies from the U.S. and Canada have developed water-quality benchmarks for 23 chemicals analyzed in this study [10]. Maximum concentrations of CECs detected in environmental samples were compared to the lowest chronic water quality benchmarks available (regardless of intended species) to assess potential toxicity of the environmental samples. A chronic benchmark was not available for prometon so the lowest available acute benchmark was used for comparison.

Environmental sediment data were compared to sediment quality guidelines established by the Wisconsin Department of Natural Resources [38] to assess the potential for risk to benthic invertebrates. Sediment quality guidelines were available for 11 of the chemicals analyzed in this study. The guidelines were predominately for PAHs but also included diethyl phthalate, phenol, and 1,4-dichlorobenzene. The maximum environmental concentration of each CEC at a given site was compared to a lower (threshold effect concentration, TEC), middle (middle effect concentration, MEC), and upper (probable effect concentration, PEC) concentration at which adverse effects to benthic-dwelling organisms are predicted to be unlikely, midway between TEC and PEC, and probable, respectively. These benchmarks focus only on potential toxicity to benthic invertebrates and do not represent bioaccumulative abilities or other organisms.

## Results and discussion

### Presence of CECs in U.S. Great Lakes tributaries

A total of 32 different CECs were frequently detected (≥30%) in water samples (Table 1) representing a variety of chemical classes including pharmaceuticals, steroid hormones, pesticides, and flame retardants. Chemicals detected in <30% of all water samples are presented in S2 Table. Maximum concentrations of the most frequently detected CECs ranged from 0.0284 (indole) to 72.2 (cholesterol) μg/L. Pharmaceuticals represented 44% of the most frequently detected CECs with maximum concentrations ranging from 0.114 (meprobamate) to 33.6 (metformin) μg/L (Table 1). Cholesterol was the only CEC detected in 100% of water samples. Maximum concentrations of cholesterol, metformin, and tris (2-butoxyethyl) phosphate were the highest of all CECs included in the study (72.2, 33.6, and 38.7 μg/L, respectively; Table 1).

In general, concentrations of CECs in water were relatively low and in ranges reported elsewhere [39–41]. However, maximum concentrations of some chemicals exceeded effects levels. For example, metformin was detected at concentrations >10 μg/L in three water samples, which has been shown to elicit increased vitellogenin mRNA expression [42]. Furthermore, intersex and reduced fecundity has been observed in *Pimephales promelas* exposed to 40 μg/L metformin [43], only slightly above the maximum concentration observed in this study (33.6 μg/L; Table 1). Tris(2-butoxyethyl) phosphate, which had a maximum water concentration of 38.7 μg/L (Table 1), can induce molecular-level effects altering protein metabolism in *Daphnia magna* at concentrations as low as 14.7 μg/L [44] and hormone synthesis in *Danio rerio* exposed to 2 μg/L [45]. This highlights only a couple chemicals for which data exist that describe effects.

Total CEC concentrations in water (based on maximum concentration of all chemicals detected at a given site) ranged from 0.32 (SCR-3) to 219 (CHI-112) μg/L (S3 Table). The greatest total concentrations were observed at CHI-112 (219 μg/L) and CHI-36 (111 μg/L) on the North Shore Channel of the Chicago River (hereafter referred to as ‘North Shore Channel’). On average, industrial chemicals, ‘other’ chemicals, pharmaceuticals, and sterols comprised >50% of total CEC concentrations in water (Figs A-K in S3 File). Different chemical signatures were observed among river basins influenced by different land uses. For example, sites with more urban influence (e.g. North Shore Channel, Clinton, Cuyahoga) typically exhibited a prominent pharmaceutical and flame retardant signature compared to sites with more agricultural influences (e.g. Grand, Kewaunee). Sterols were ubiquitous across all sites and generally represented a large proportion of total CEC concentrations in water (65%, on average).

Estimated EEQ in water ranged from 0 (several St. Clair sites and Grand-4) to 28 (RAQ-2) ng/L (S3 Table). Steroid hormones contributed the least to EEQ in water. Despite low detection frequencies, maximum concentrations for several alkylphenols exceeded 1 μg/L (S2 Table), contributing substantially to the overall EEQ. The EEQ for all St. Clair River sites was <1 ng/L, indicating the relatively few chemicals and low concentrations of estrogenic chemicals detected along this river reach. Relatively high EEQ was consistently observed at Little Calumet and North Shore Channel sites; all but one site were >10 ng/L, well within concentrations reported to elicit effects such as induction of vitellogenin in mussels [47], and fish [48–49]. The EEQ at all sites on the Cuyahoga and Raquette Rivers were >1 ng/L, also at levels expected to elicit effects to aquatic biota [47–49]. The EEQ tended to increase directly downstream of WWTP effluent discharges (e.g. KWE-5, RAQ-2). These increases are in agreement with observations of the estrogenic properties of WWTP effluent [49,50].

A total of 28 different chemicals were frequently detected (≥30%) in sediment samples (Table 1) representing a variety of chemical classes including fecal indicators, PAHs, steroid hormones, and fragrances. Chemicals detected in <30% of sediment samples are presented in S2 Table. Maximum concentrations of the most frequently detected chemicals ranged from 1.75 (diphenhydramine) to 20,800 (fluoranthene) μg/kg. About one-third of the frequently detected chemicals in sediment were PAHs with maximum concentrations often >1,000 μg/kg. Consequently, these chemicals represented a large proportion of total CEC concentrations in sediment (Figs A-K in S4 File). Other frequently detected CECs included fecal indicators, industrial chemicals, and steroid hormones. The two fecal indicators, indole and 3-methyl-1*H*-indole, were detected in 100 and 91% of all sediment samples, respectively. Four steroid hormones were detected in at least 30% of sediment samples, whereas none were frequently detected in surface-water samples. One pharmaceutical was frequently detected in sediment samples. However, chemical recovery of pharmaceuticals in sediment samples was often below 30%, potentially underestimating the presence and magnitude of pharmaceuticals.

Total CEC concentrations in sediment (based on maximum concentration of all chemicals at a given site) ranged from 0 (OSW-3) to 47,248 (CLI-4) μg/kg (S3 Table). Sterols were the most abundant chemical class in sediment samples, representing, on average, 42% of total CEC concentrations. Polycyclic aromatic hydrocarbons and fecal indicators represented, on average, 34 and 7% of total sediment concentrations, respectively. A less pronounced difference in chemical signatures among river basins was observed in sediment samples compared to that seen for water (Figs A-K in S4 File). Industrial chemicals were more prevalent in urban-influenced rivers compared to agricultural. Similar to water samples, sterols were ubiquitous in sediments across the U.S. Great Lakes basin and represented a relatively large proportion of total chemical concentrations (41%, on average).

Despite CECs being detected frequently in river and lake sediments [51–53], significant knowledge gaps exist regarding the implications of sediment-bound CECs. Specifically, there is limited information available regarding the re-release of CECs to overlying water and exposure pathways to fish and wildlife. These knowledge gaps pertaining to CEC exposure pathways in sediments may impact wildlife risk assessments. For example, while the effects of aqueous exposure of hormones to vertebrates are well studied [54–56], not much is known regarding the effects these chemicals have on sediment-dwelling invertebrates, such as mussels and other macroinvertebrates. In addition, there is very little data focused on the risk posed to predators such as bottom-feeding fish or insectivorous birds that forage on sediment-dwelling organisms.

Estimated EEQ in sediment ranged from 0 (several sites) to 25,864 (CHI-76) ng/kg (S3 Table). Similar to EEQ in water, alkylphenols contributed the most to EEQ in sediment, with the exception of the RAQ sites, where bisphenol A had a relatively high contribution. The EEQs for all Cuyahoga River sites were <1,000 ng/kg; the most consistent pattern of all the sampled tributaries. All the Tinkers Creek sites generally had low EEQ (<500 ng/kg), except for TIC-1, the most upstream site (19,389 ng/kg), which is surprising given the number of WWTPs along the sampled reach. Again, no apparent downstream pattern was observed in the chemical composition of sediment samples. Additionally, the presence of a WWTP did not always correspond to an increase in estrogenicity at the nearest downstream site as was observed in water samples. The relatively consistent patterns seen in sediment CEC concentrations and EEQ demonstrate that sediment dwelling organisms may be consistently exposed to these chemicals no matter their location, unlike pelagic species that may be exposed more intermittently.

Of the 60 chemicals frequently detected in water and sediment, only eight were frequently detected in both matrices. Though differences in chemicals present in water versus sediment are not unexpected, differences observed in this study demonstrate the importance of understanding the chemical signature of the whole environment and not only water, as many studies are focused [1,57,58]. Many factors play a role in the fate and transport of CECs in the environment. For example, the K_ow_ provides an indication of whether a chemical will more likely partition to water or sediment. Generally, the K_ow_ of the most frequently detected chemicals in this study explain the differences between water and sediment detections. A majority of the most frequently detected chemicals in water have K_ow_<3, while those in sediment have K_ow_ >3. However, in-stream processes (e.g. hydrology, organic matter content, etc.) can affect the actual partitioning of CECs in the environment. Additionally, K_ow_ values are based on an environment assumed to be at equilibrium. Most of our sample sites were located in dynamic stream environments where factors such as hydrology and other physical stream process are in flux. Therefore, it is hard to make generalizations regarding how K_ow_ might affect any given chemical across the Great Lakes basin. However, this data set could provide a basis for further investigation of the processes controlling CEC transport and fate in tributaries to the Great Lakes.

### Cluster analysis

**Fig 2:**
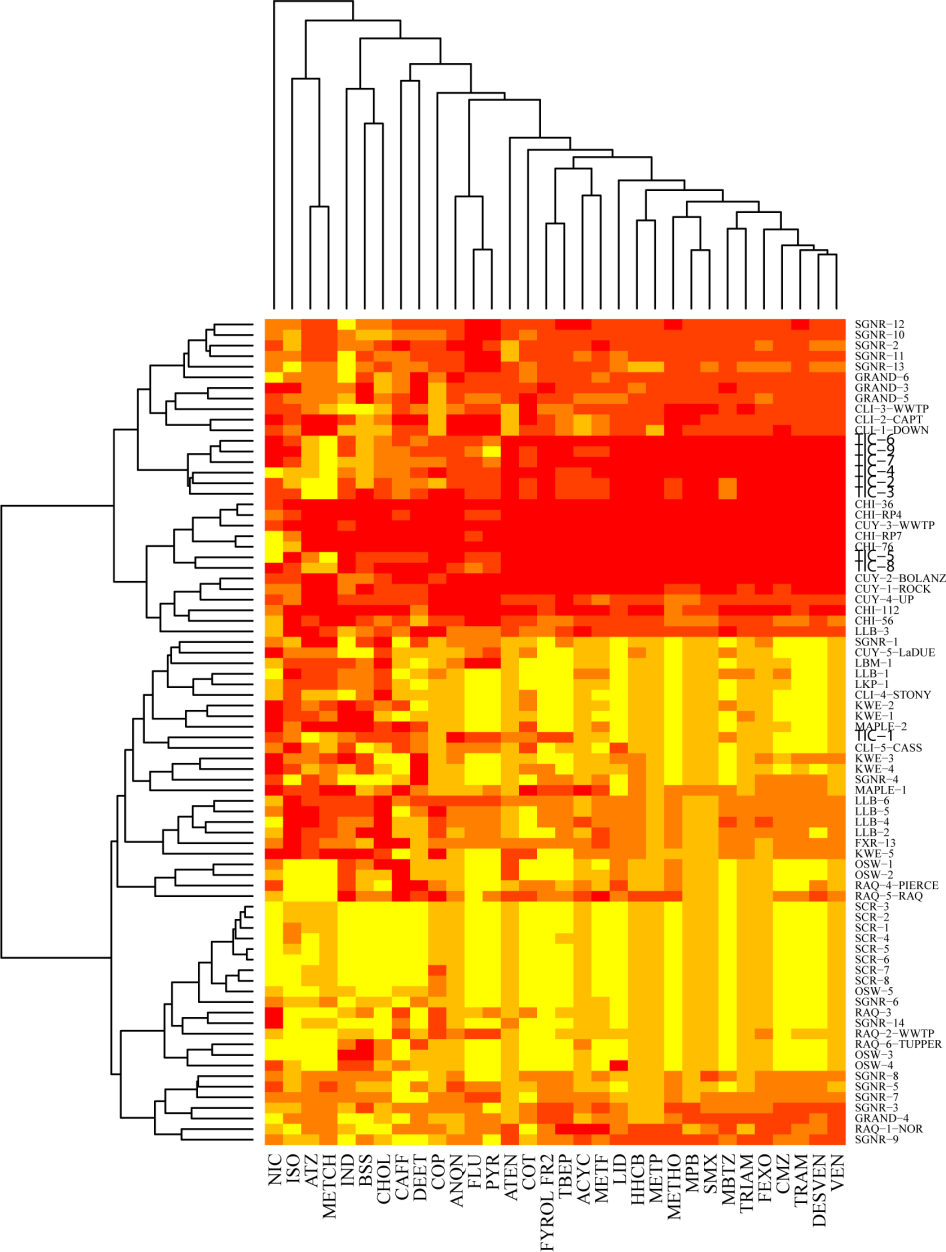
Heatmap of two-way cluster analysis performed on rank-transformations of maximum concentrations detected per site (only includes chemicals detected ≥30% of water samples). Data ranks are represented by color; lighter colors correspond to lower ranks. NIC, nicotine; ISO, isophorone; ATZ, atrazine; METCH, metolachlor; IND, indole; BSS, β-sitosterol; CHOL, cholesterol; CAFF, caffeine; DEET, N,N-Diethyl-*meta*-toluamide; COP, 3β-coprostanol; ANQN, 9,10-anthraquinone; FLU, fluoranthene; PYR, pyrene; ATEN, atenolol; COT, cotinine; FYROL FR2, tris (dichloroisopropyl) phosphate; TBEP, tris(2-butoxyethyl) phosphate; ACYC, acyclovir; METF, metformin; LID, lidocaine; HHCB, hexahydrohexamethyl cyclopentabenzopyran; METP, metoprolol; METHO, methocarbamol; MPB, meprobamate; SMX, sulfamethoxazole; MBTZ, methyl-1*H*-benzotriazole; TRIAM, triamterene; FEXO, fexofendadine; CMZ, carbamazepine; TRAM, tramadol; DESVEN, desvenlafaxine; VEN, venlafaxine.

**Fig 3:**
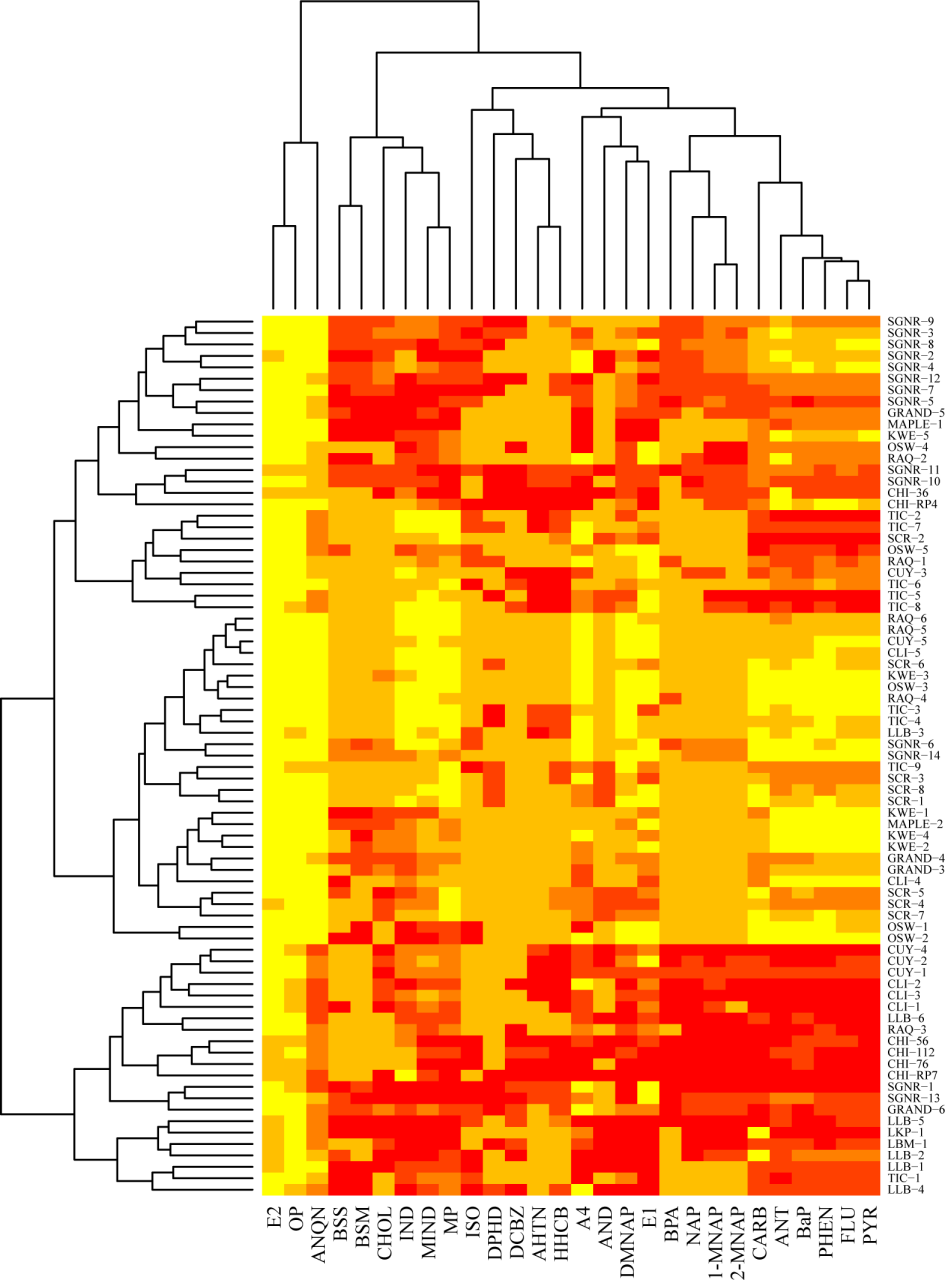
Heatmap of two-way cluster analysis performed on rank-transformed sediment data (only includes chemicals detected ≥30% of sediment samples). Data ranks are represented by color; lighter colors correspond to lower ranks. E2, 17β-estradiol; OP, 4-*tert*-octylphenol; ANQN, anthraquinone; BSS, β-sitosterol; BSM, β-stigmastanol; CHOL, cholesterol; IND, indole; MIND, 3-methyl-indole; MP, *p*-cresol; ISO, isophorone; DPHD; diphenhydramine; DCBZ, 1,4-dichlorobenzene; AHTN, acetyl hexamethyl tetrahydronaphthalene; HHCB, hexahydrohexamethyl cyclopentabenzopyran; A4, 4-androsterne-3,17-dione; AND, *cis*-androsterone; DMNAP, 2,6-dimethylnaphthalane; E1, estrone; BPA, bisphenol A; NAP, naphthalene; 1-MNAP, 1-methylnaphthalene; 2-MNAP, 2-methylnaphthalene; CARB, carbazole; ANT, anthracene; BaP, benzo(a)pyrene; PHEN, phenanthrene; FLU, fluoranthene; PYR, pyrene.

Cluster analysis of the CECs measured in water and sediment revealed patterns of co-occurring chemicals (Fig 2 and Fig 3) that might be expected based on basic land use information such as presence of WWTP and land cover. For example, pharmaceuticals were often identified in surface water of rivers with several WWTP discharges along the sampled reach (Tinkers Creek and Cuyahoga River). Because WWTPs are a major source of pharmaceuticals to the environment [59], large mixtures of pharmaceuticals are expected to co-occur downstream from these sources. Several CECs did not cluster with any others in both matrices [β-sitosterol (BSS), DEET, 3β-coprostanol (COP), and caffeine (CAFF) in water; cholesterol (CHOL), isophorone (ISO), 4-androstene-3,17-dione (A4) in sediment] demonstrating the ubiquitous nature of these CECs in the U.S. portion of the Great Lakes Basin. Several small clusters were identified representing pesticides [metolachlor (METCH) and atrazine (ATZ); Fig 2] and flame retardants [tris(2-chloroethyl) phosphate (FYROL) and tris(butoxyethyl) phosphate (TBEP)] in water, as well as, fragrances [acetyl hexamethyl tetrahydronaphthalene (AHTN) and hexahydrohexamethyl cyclopentabenzopyran (HHCB)] and PAHs in sediment. The plant sterols co-occurred in sediments from both agricultural and urban sites indicating that there are various sources of these chemicals to the environment.

Surface-water sites from a specific river generally clustered together, as well as with sites from other rivers with similar contaminant sources. For example, Little Calumet and Cuyahoga sites clustered with a couple of Tinker’s Creek sites based on their chemical signatures (several pharmaceuticals and flame retardants), indicating the heavy influence of WWTPs [59,60] in these systems (Fig 2). Similarly, the St. Clair and Oswegatchie sites clustered together; these rivers typically had relatively few chemical detections in water samples. Sites located on rivers in more agriculturally influenced watersheds (e.g. LLB, KWE) tended to cluster together with similar chemical signatures such as atrazine and metolachlor. Almost all of the chemicals included in the cluster analyses were detected at relatively high concentrations in the Little Calumet and North Shore Channel sites. These rivers have a highly complex chemical signature indicative of the heavy urban influence in comparison to other sites. The heatmap also highlights the relatively simple chemical signatures (i.e. few chemicals and relatively low concentrations) among the sites on the St. Clair River which most likely reflects the influence of Lake Huron water diluting urban inputs along the sampled reach.

Sediment sites from the same river also often clustered together indicating similar chemical signatures among sites in the same river basin in sediment samples. Cuyahoga, Clinton, North Shore Channel, and Little Calumet display CEC signatures similar to each other, and are more indicative of heavy urban influence with relatively high concentrations of PAHs and other industrial chemicals. The middle section of Fig 3 represents sites located in the upper to middle reaches of the sampled rivers and generally had relatively little direct anthropogenic influence. These sites have relatively moderate concentrations of most chemicals. The chemicals with relatively high concentrations at these less-influenced sites mostly represent naturally-occurring chemicals such as plant sterols, indole, and estrone.

### Comparisons to benchmarks

Water-quality benchmarks were exceeded for at least one chemical at 35 (45%) sites representing 10 (83%) river basins (S4 Table). Water quality benchmarks for seven chemicals [4-nonylphenol, anthracene, benzo(a)pyrene, bis(2-ethylhexyl) phthalate, dichlorvos, fluoranthene, and pyrene] were exceeded at least once (Table 2). While most of the exceeded benchmarks represent PAHs (chemicals known to be present in the sampled areas because of past or current activities), other chemical classes are represented such as a detergent metabolite, plasticizer, and pesticide. Benchmarks for benzo(a)pyrene and pyrene were exceeded more often than any other chemicals. Site SGNR-10 exceeded benchmarks for five chemicals [benzo(a)pyrene, bis(2-ethylhexyl) phthalate, dichlorvos, fluoranthene, and pyrene], the most of any site. In contrast, eight of the 14 Saginaw River sites did not exceed benchmarks for any chemical. Finally, benchmarks were not exceeded in any of the surface-water samples collected from the Kewaunee, Maple, or St. Clair Rivers.

**Table 2:** Percent of sampled sites in U.S. tributaries to the Great Lakes exceeding water-quality benchmarks. Table includes only those chemicals with at least one exceedance.

River	Number of sites sampled	4-Nonylphenol	Anthracene	Benzo[a]pyrene	Bis (2-ethylhexyl) phthalate	Dichlorvos	Fluoranthene	Pyrene
Water Quality Benchmark^a^ (micrograms per liter)		1.00	0.01	0.01	0.30	0.01	0.04	0.03
Fox	9	--	100	50	--	--	29	29
Kewaunee	5	0	--	--	--	--	--	--
Little Calumet	3	0	100	100	--	--	100	100
North Shore Channel of the Chicago River	3	0	50	100	--	--	100	100
Clinton	5	--	50	100	--	--	75	75
Grand/Maple	6	0	0	33	--	--	20	20
Saginaw	14	--	0	63	100	100	60	75
St. Clair	8	--	--	--	--	--	--	--
Cuyahoga	5	0	60	80	--	--	60	80
Tinkers	9	33	25	50	--	100	44	75
Oswegatchie	5	0	--	--	100	--	--	--
Raquette	6	0	0	100	100	--	0	25
Basin Wide	78	1	17	40	6	9	39	44

--, not detected

^a^Baldwin et al. [10]

Sediment samples collected from several sites exceeded at least the TEC sediment quality guideline for all chemicals (for which guidelines exist), with the exception of diethyl phthalate (Table 3, S5 Table). Diethyl phthalate was only detected in the Saginaw River and one site in the Grand River; environmental concentrations were consistently lower than the TEC. Phenol was detected at 16 sites and environmental concentrations always exceeded the PEC. Multiple sites within Little Calumet, North Shore Channel, Clinton River, Cuyahoga River, Little Lake Butte des Morts, Saginaw River, and Tinkers Creek exceeded the PEC for at least half of the chemicals for which sediment quality guidelines exist. Furthermore, at least one site within each sampled river exceeded the PEC for one or more chemicals. Only two sites (KWE-1 and KWE-2) did not exceed any sediment benchmark, indicating that the study chemicals are ubiquitous throughout the sampled tributaries to the Great Lakes and most likely at concentrations above which effects to benthic-dwelling invertebrates are likely to occur.

**Table 3:** Percent of sampled sites in U.S. tributaries to the Great Lakes exceeding sediment quality guidelines provided in WIDNR [38]. Table includes only those chemicals with at least one exceedance.

**A**																
**River**	**Number of sites sampled**	**1,4-Dichlorobenzene**			**2-Methylnaphthalene**			**Anthracene**			**Benzo[a]pyrene**			**Fluoranthene**		
		**TEC**	**MEC**	**PEC**	**TEC**	**MEC**	**PEC**	**TEC**	**MEC**	**PEC**	**TEC**	**MEC**	**PEC**	**TEC**	**MEC**	**PEC**
Sediment Quality Guideline (micrograms per kilogram)		31	60.5	90	20.2	111	201	57.2	451	845	150	800	1,450	423	1,327	2,230
Fox	8	25	0	0	100	80	80	88	63	63	100	100	86	100	100	88
Kewaunee	5	--	--	--	--	--	--	--	--	--	0	0	0	100	0	0
Little Calumet	3	100	67	67	100	100	100	100	100	100	100	100	100	100	100	100
North Shore Channel of Chicago River	3	66	33	0	66	33	0	100	100	100	67	67	67	100	100	100
Clinton	5	100	0	0	100	50	50	75	75	75	60	60	60	75	75	75
Grand/Maple	6	0	0	0	0	0	0	40	20	20	67	50	20	100	80	60
Saginaw	14	57	0	0	29	7	0	30	10	10	67	67	42	71	71	57
St. Clair	8	--	--	--	--	--	--	13	13	13	38	13	13	88	88	38
Cuyahoga	5	--	--	--	100	33	33	75	50	50	75	75	75	75	75	75
Tinkers	9	0	0	0	100	50	50	63	63	50	88	75	63	89	78	78
Oswegatchie	5	0	0	0	100	100	100	50	50	50	100	50	50	100	100	67
Raquette	6	0	0	0	100	100	50	50	25	25	60	60	40	80	60	60
Basin Wide	77	48	13	13	65	41	32	54	42	40	69	60	48	86	80	67
**B**																
**River**	**Number of sites sampled**	**Naphthalene**			**Phenanthrene**			**Phenol**			**Pyrene**					
		**TEC**	**MEC**	**PEC**	**TEC**	**MEC**	**PEC**	**TEC**	**MEC**	**PEC**	**TEC**	**MEC**	**PEC**			
Sediment Quality Guideline (micrograms per kilogram)	176	369	561	204	687	1,170	4,200	8,100	12,000	195	858	1,520				
Fox	8	100	100	100	100	100	100	--	--	--	100	88	88			
Kewaunee	5	--	--	--	100	0	0	100	100	100	100	100	100			
Little Calumet	3	100	100	100	100	100	100	100	100	100	100	67	67			
North Shore Channel of Chicago River	3	50	50	0	100	100	100	--	--	--	100	100	100			
Clinton	5	67	67	33	100	100	100	--	--	--	75	75	75			
Grand/Maple	6	100	0	0	60	60	40	100	100	100	100	80	60			
Saginaw	14	36	14	14	77	62	54	100	100	100	71	71	57			
St. Clair	8	--	--	--	100	80	60	--	--	--	88	63	38			
Cuyahoga	5	100	33	33	100	100	100	--	--	--	75	75	75			
Tinkers	9	--	--	--	100	86	86	--	--	--	89	78	78			
Oswegatchie	5	--	--	--	100	100	100	--	--	--	100	67	67			
Raquette	6	100	50	50	60	60	60	--	--	--	80	60	60			
Basin Wide	77	67	45	39	88	79	73	100	100	100	85	72	66			

TEC, threshold effect concentration; MEC, midpoint effect concentration; PEC, probable effect concentration; --, no detected

Although benchmarks were available for only a limited number of chemicals, this study can still provide an initial vulnerability assessment useful for resources management. For example, benzo(a)pyrene water-quality benchmarks and sediment quality guidelines were often exceeded (>50% of rivers) in samples. In addition to understanding the potential exposure pathways, understanding other chemicals that co-occur with those for which benchmarks exist is equally important. For example, cluster analyses show that benzo(a)pyrene (and PAHs in general) often co-occurred at urban sites where other chemicals such as pharmaceuticals often occurred. Some studies have shown additive effects of exposure to PAHs and metals [61,62], however little is known regarding interactions between PAHs and organic chemicals such as CECs. If organisms are already being exposed to a chemical at levels exceeding or near a benchmark and other chemicals with unknown benchmarks but similar modes of action are being detected in the same location, the risk to organisms may be more than would have been originally predicted with the single chemical benchmark.

## Conclusions

This study presents the first basin-wide analysis of CECs in water and sediments of U.S. tributaries to the Great Lakes. These data provide background information about CECs and other chemicals of interest in the sampled reaches. This information can be used to develop tools to inform management decisions aimed at reducing aquatic biota exposure in the Great Lakes Basin. Study results provide a framework for further work to better understand the effects of CEC mixtures on aquatic biota and the relationship between landscape and CEC presence in surface waters and sediments. We present multiple areas in which further information is needed to more fully understand the potential impacts to fish and wildlife resources, including chemical interactions and the implications of chemical transport between sediment and water. Furthermore, results of this study can be used to guide detailed assessments, such as studies to determine degradation rates and assimilative capacity of chemicals, or to identify CEC mixtures that likely pose a threat to ecosystem health.

## Supporting information

S1 Table Surface-water and sediment sites sampled for analysis of a broad suite of contaminants of emerging concern in U.S. tributaries to the Great Lakes, 2013–14. (XLSX)

S2 Table Chemicals detected in <30% of all surface-water and sediment samples collected from U.S. tributaries to the Great Lakes, 2013–14. (XLSX)

S3 Table Concentrations of chemical classes, total site concentrations, and calculated estradiol equivalents (EEQ) (based on maximum concentration detected in any sample at a given site) in water and sediment samples collected in U.S. tributaries to the Great Lakes, 2013–4. Units are micrograms per liter for water, micrograms per kilogram for sediment, and nanograms per liter (or kilogram) for EEQ.
(XLSX)

S4 Table Ratio of maximum water concentrations to water-quality benchmarks for samples collected from U.S. tributaries to the Great Lakes, 2013–14. Quotients greater than or equal to one are highlighted in bold, red type.
(XLSX)

S5 Table Ratio of maximum environmental sediment concentrations to sediment quality guidelines (38) for samples collected from U.S. tributaries to the Great Lakes, 2013–14. Quotients greater than or equal to one are highlighted in bold, red type.
(XLSX)

S1 File Example R code used to produce heatmaps. (PDF)

S2 File Maps of specific study site locations. (PDF)

S3 File Relative concentrations (based on maximum concentrations reported in S3 Table) of chemical classes in water samples from U.S. tributaries to the Great Lakes. Sterols excluded because they comprised a relatively large percentage of total concentrations. The difference between 100 and total percent shown in graphs represents sterols.
(PDF)

S4 File Relative concentrations (based on maximum concentrations reported in S3 Table) of chemical classes in sediment samples from U.S. tributaries to the Great Lakes. Sterols excluded because they comprised a relatively large percentage of total concentrations. The difference between 100 and total percent shown in graphs represents sterols.
(PDF)
