# Supplementary material for: Contaminants of emerging concern in tributaries to the Laurentian Great Lakes: I. Patterns of occurrence
Source: PLoS One. 2017 Sep 27;12(9):e0182868. doi: 10.1371/journal.pone.0182868 (PMC5617142; doi:10.1371/journal.pone.0182868)
Supplement: S1 File — (PDF) [file pone.0182868.s006.pdf]

Cluster Analysis R Code

Two-dimensional cluster analyses with heatmaps allow simultaneous viewing of R-mode (groups of variables) and Q-mode (groups of sites) clustering, as well as a color scale that conveys the magnitude of a number in individual cells (e.g. the concentration of a chemical at a site). We used the heatmap.2 function in the gplots package of R software (version 3.1.3) to produce heatmap diagrams.

We used default euclidian distance calculations to generate the distance matrices used for clustering.

Example R code is shown below:

```
install.packages (“gplots”) #installs gplots, which houses heatmap.2
library(gplots) #loads gplots package

pdf(“filename.pdf”) #Open pdf output device named ‘filename’

heatmap.2(data.matrix(data[,4:30]), #uses the input data set ‘data’
      labRow=data$NewFieldName, #NewFieldName is the abbreviated site
      name
      scale=”none”, #turn off default scaling
      margins=c(10,10),
      col=rev(heat.colors(6))[-1], #defines color scale, and reverses
            order so that lightest color (yellow) corresponds to lowest
            concentraitons and warmest (red) corresponds to highest
            concentrations of each variable
      trace=”none”, #turn off histogram trace
)

dev.off() #Closes pdf output device and writes pdf file
```
