# Supplementary material for: Contaminants of emerging concern in tributaries to the Laurentian Great Lakes: I. Patterns of occurrence
Source: PLoS One. 2017 Sep 27;12(9):e0182868. doi: 10.1371/journal.pone.0182868 (PMC5617142; doi:10.1371/journal.pone.0182868)
Supplement: S2 File — (PDF) [file pone.0182868.s007.pdf]

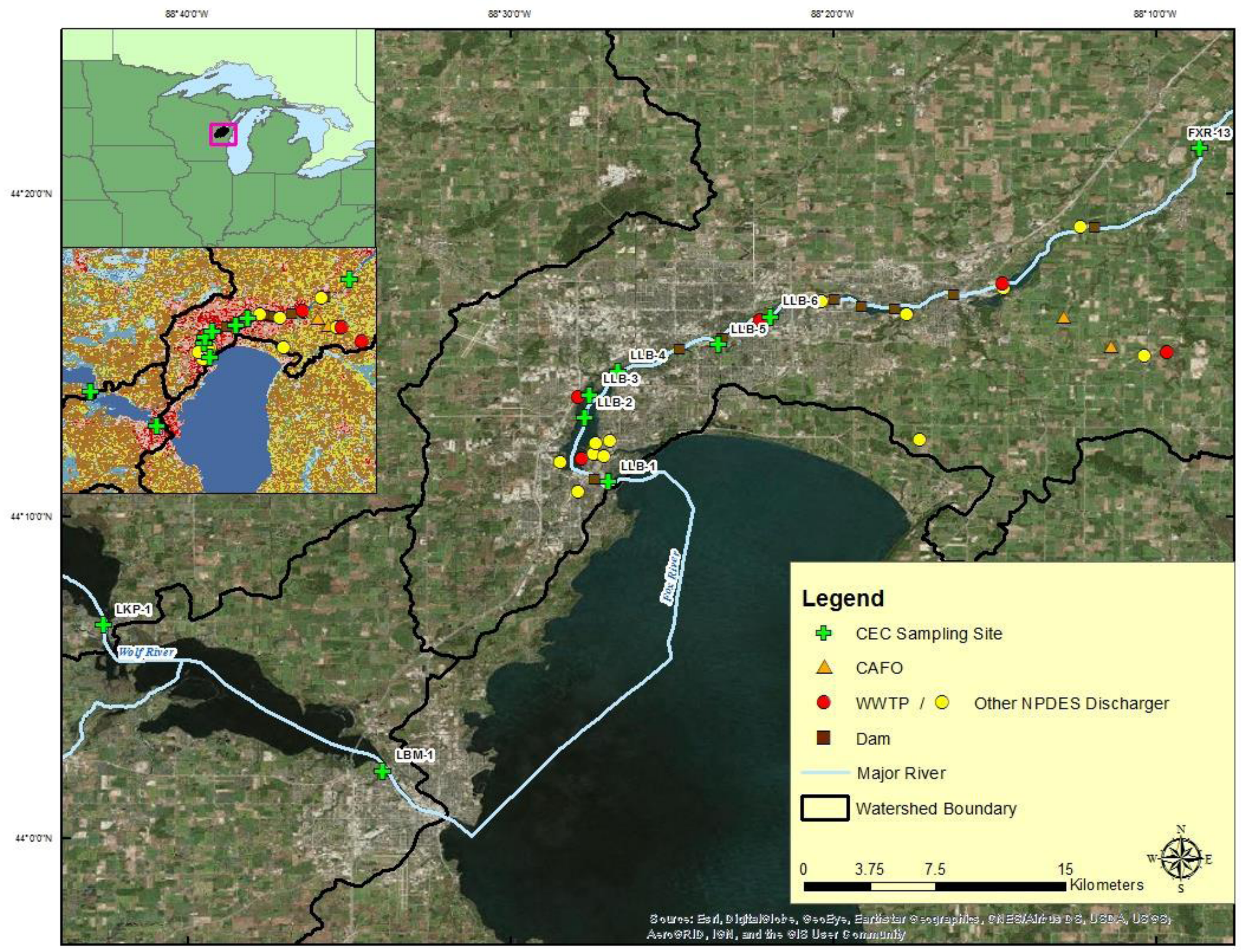

Fig A. Map of Fox River sites sampled for contaminants of emerging concern, 2013-14. River flows northeast.

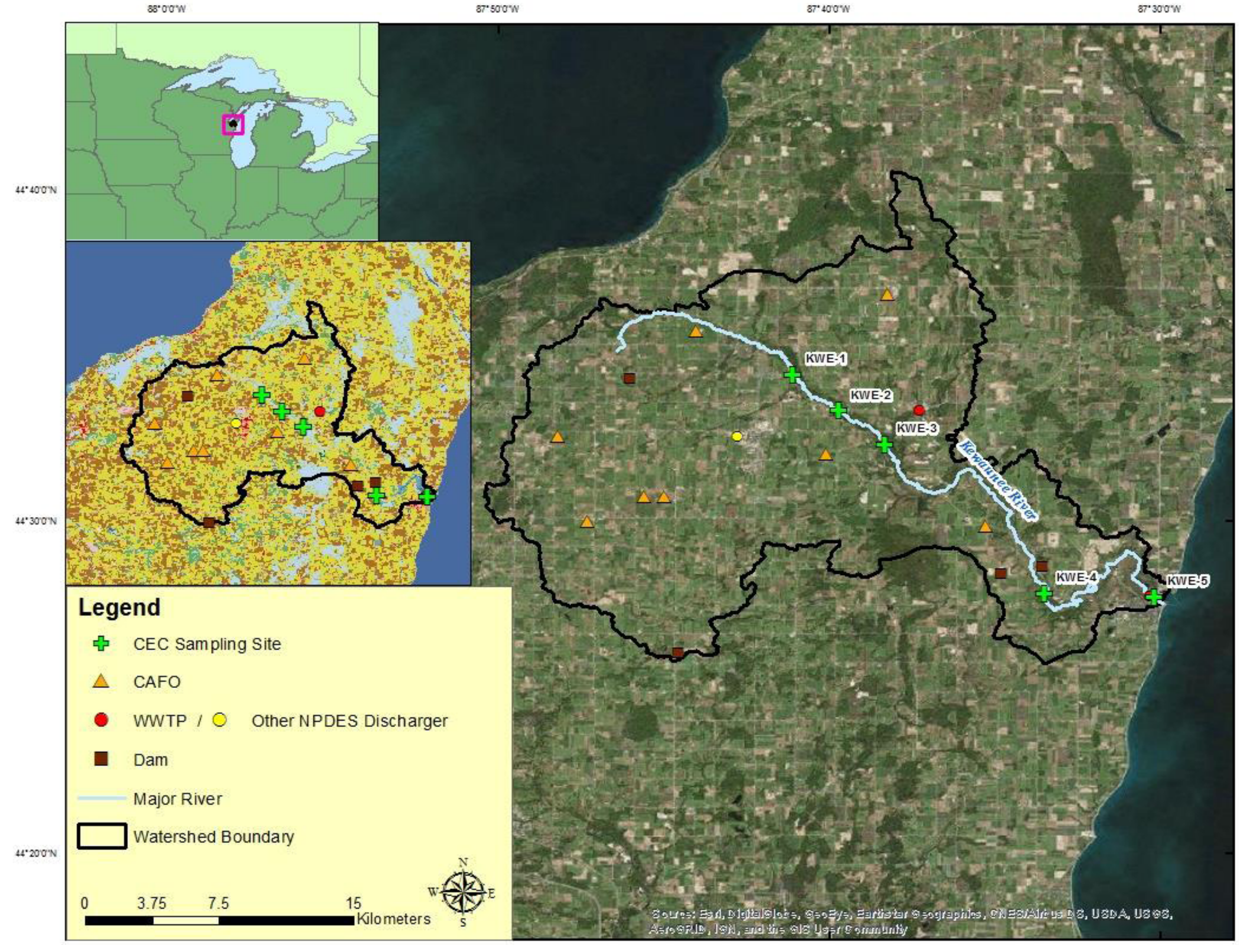

Fig B. Map of Kewaunee River sites sampled for contaminants of emerging concern, 2013-14. River flows east.

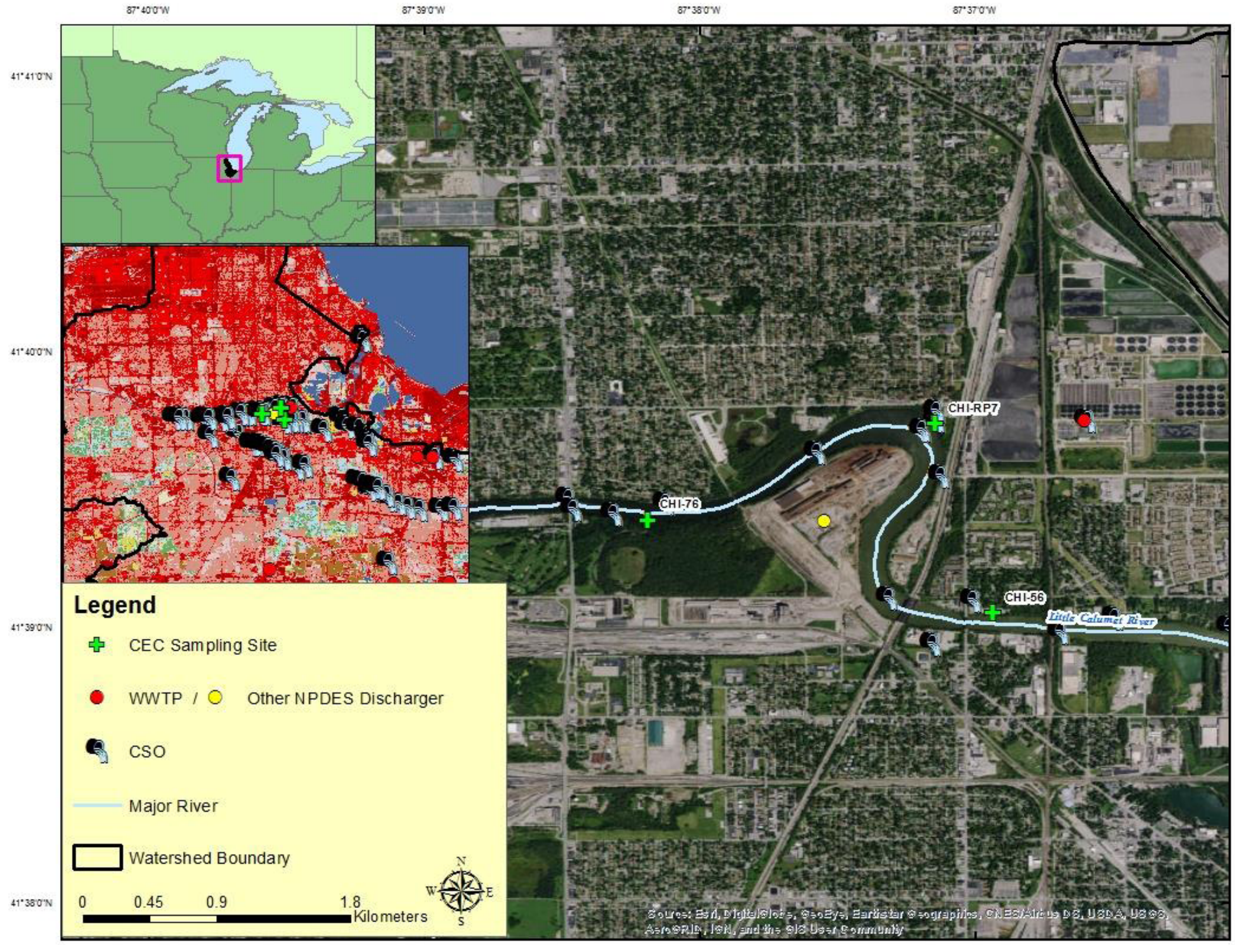

Fig C. Map of Little Calumet River sites sampled for contaminants of emerging concern in 2014. River flows west.

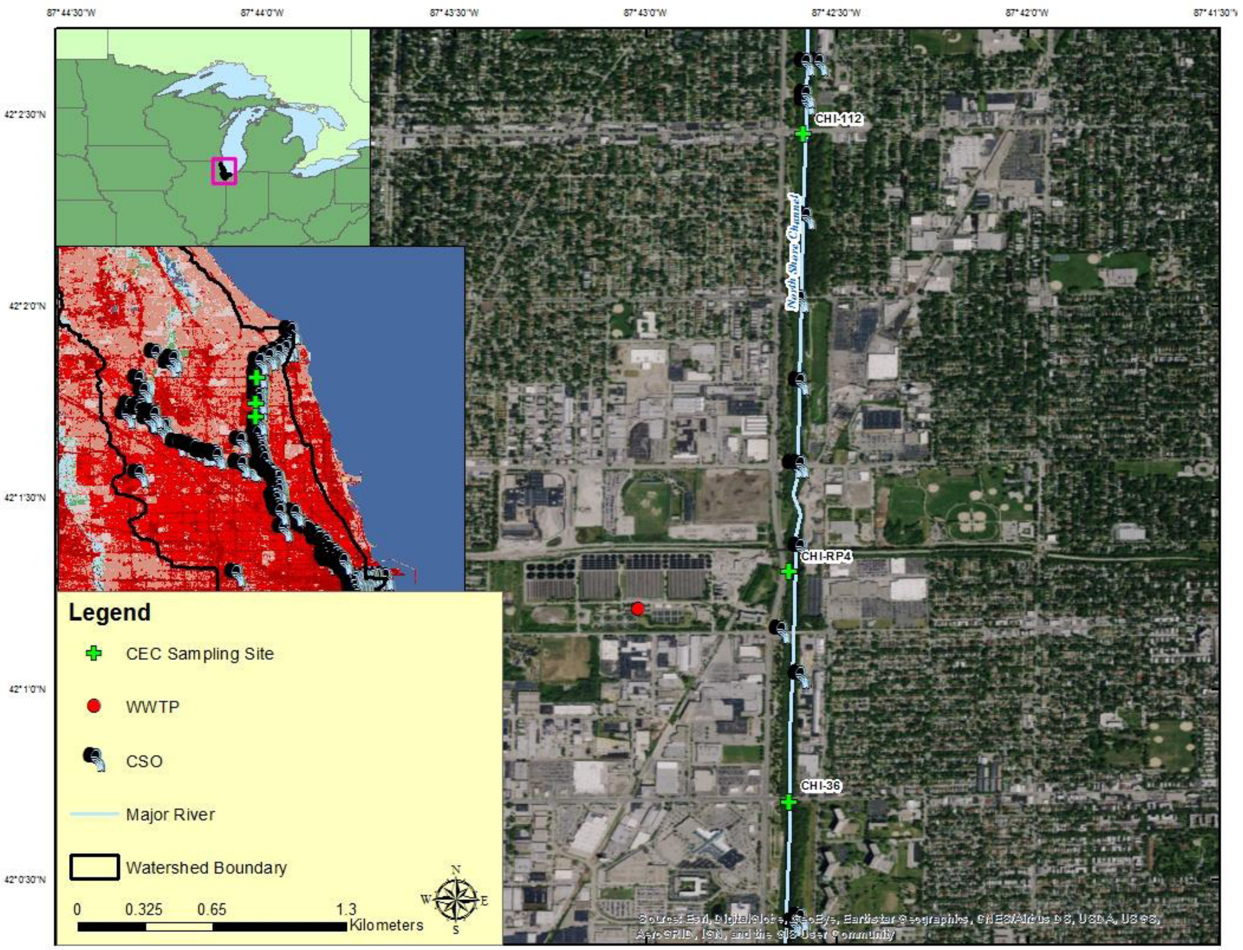

Fig D. Map of the North Shore Channel of the Chicago River sites sampled for contaminants of emerging concern in 2014. River flows south.

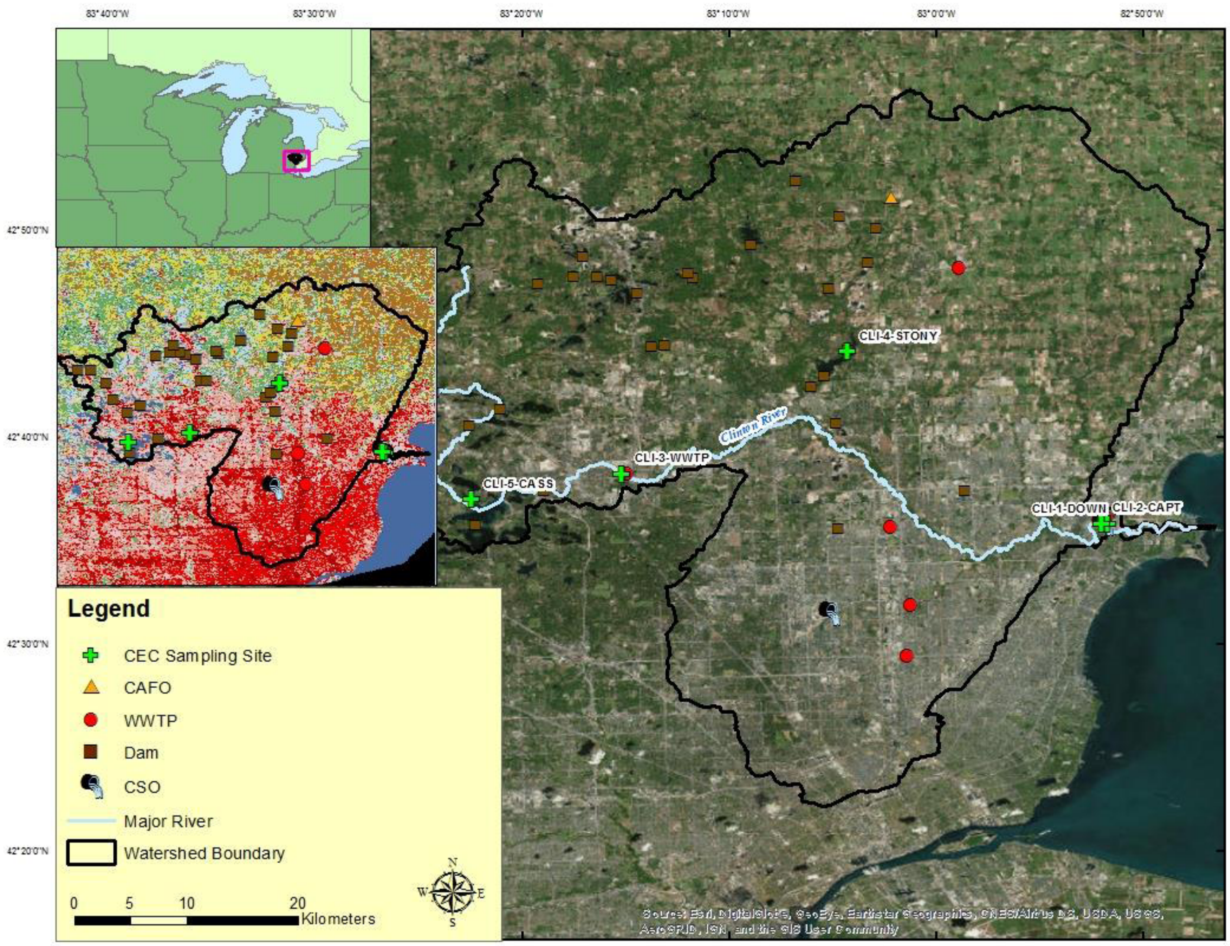

Fig E. Map of Clinton River sites sampled for contaminants of emerging concern, 2013-14. River flows east.

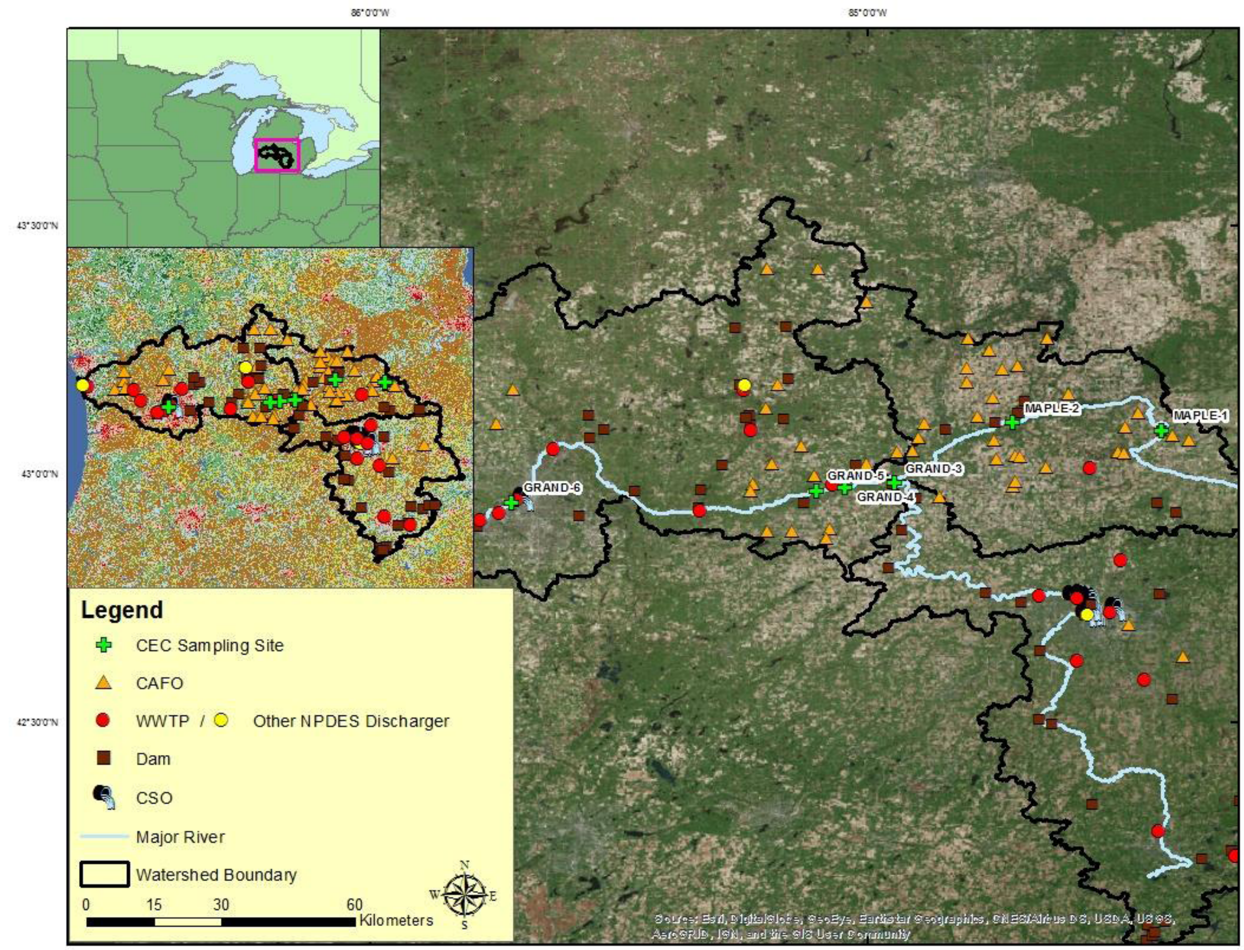

Fig F. Map of Grand and Maple River sites sampled for contaminants of emerging concern, 2013-14. Rivers flow west.

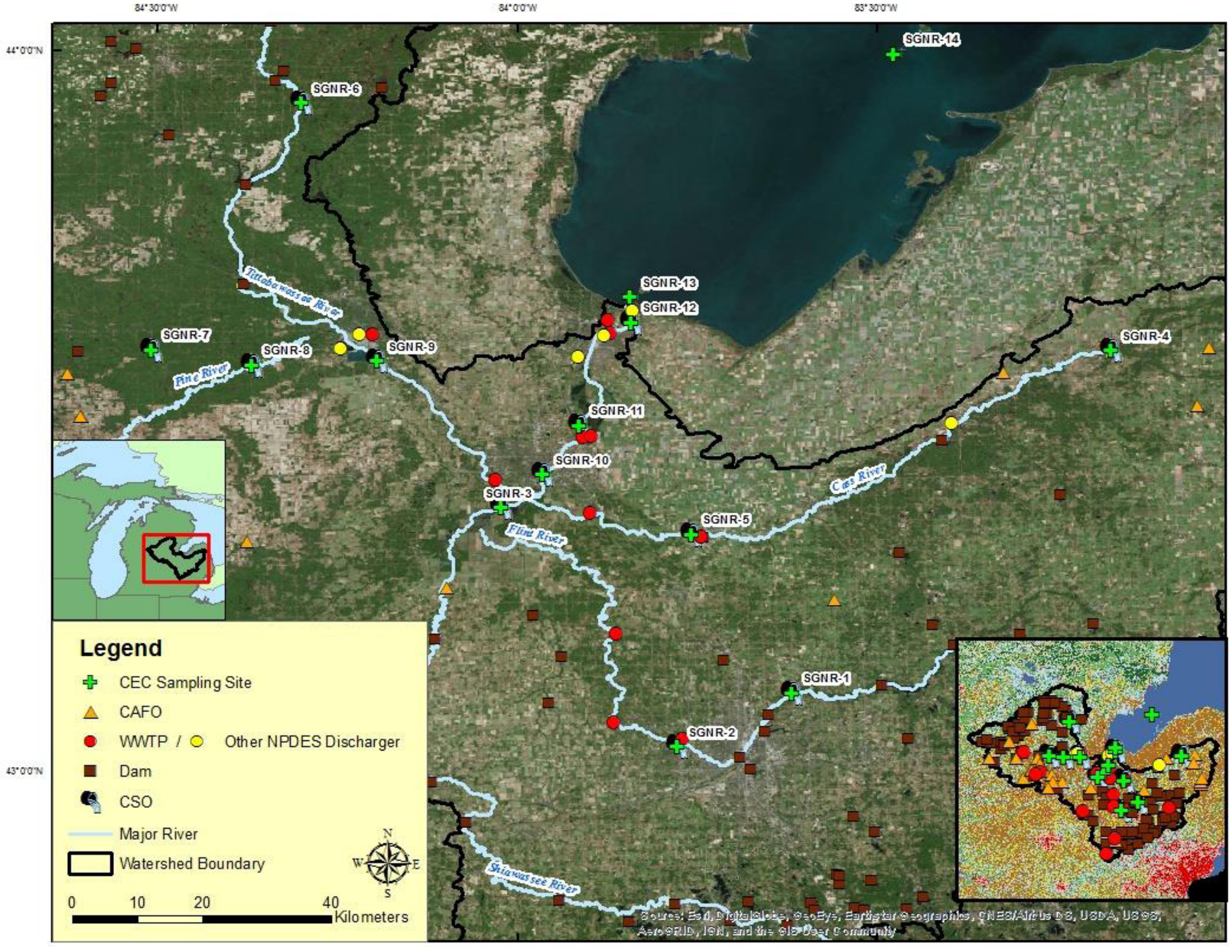

Fig G. Map of Saginaw River sites sampled for contaminants of emerging concern in 2013. River flows north.

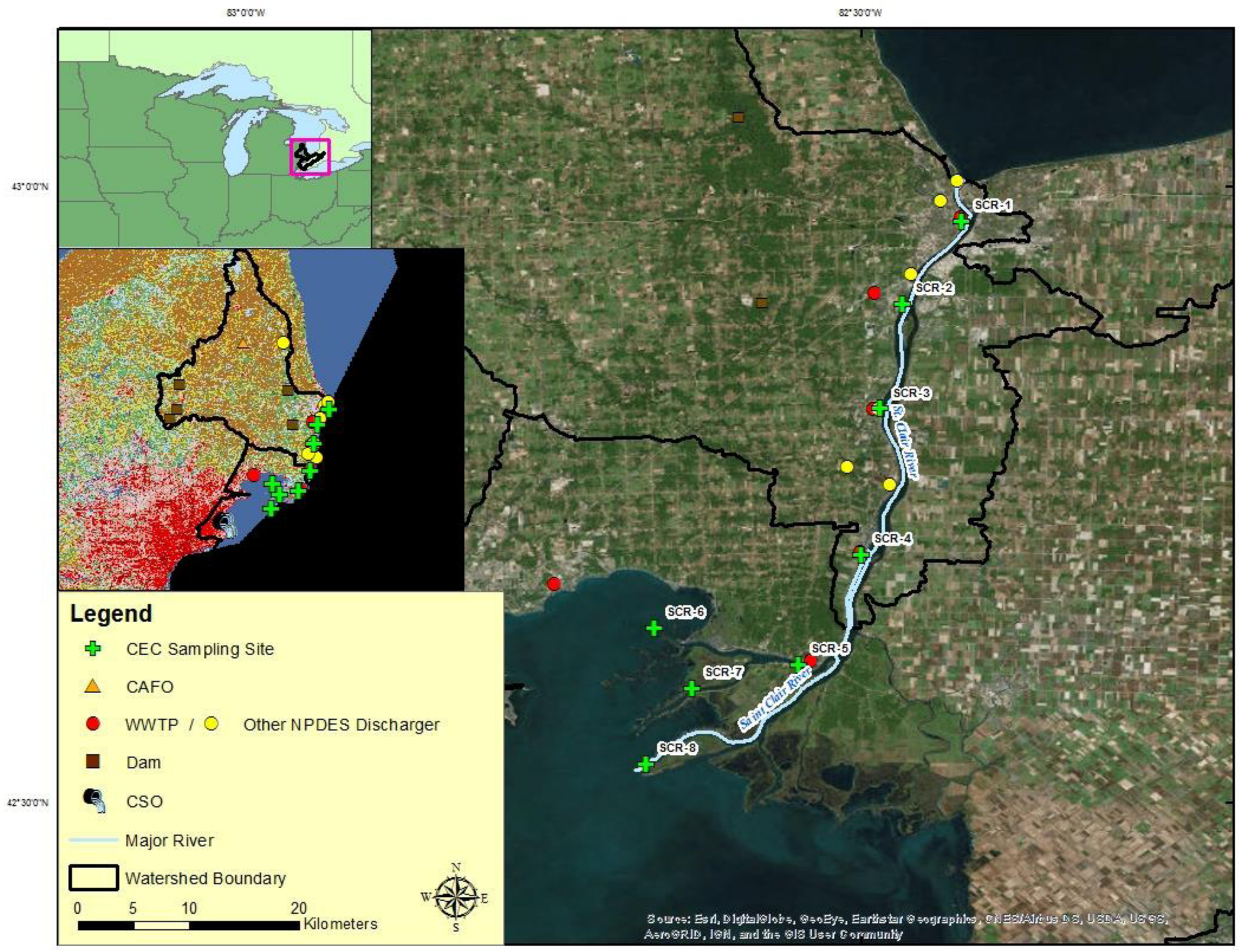

Fig H. Map of St. Clair River sites sampled for contaminants of emerging concern in 2013. River flows southwest.

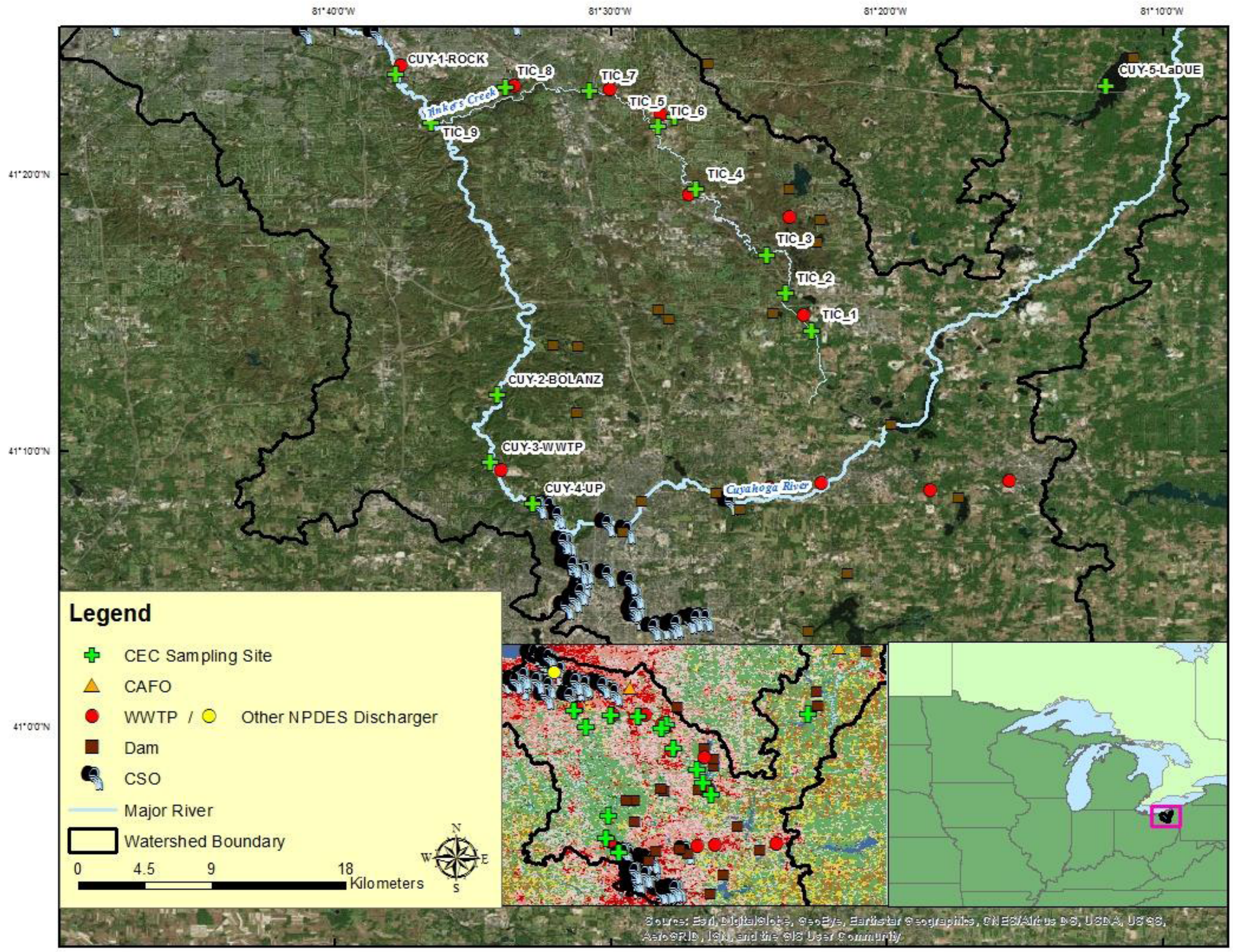

Fig I. Map of Tinkers Creek and Cuyahoga River sites sampled for contaminants of emerging concern, 2013-14. Rivers flow north.

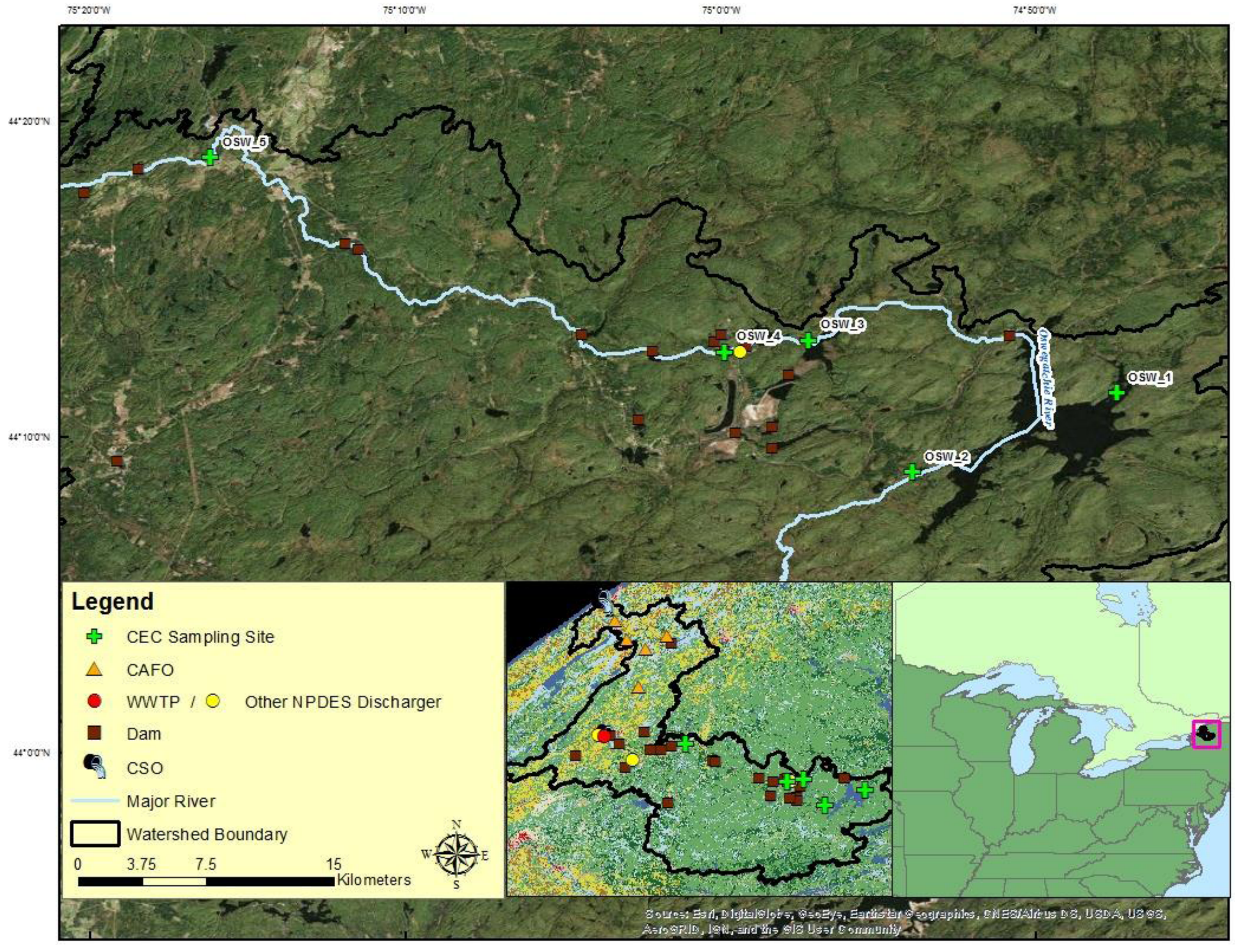

Fig J. Map of Oswegatchie River sites sampled for contaminants of emerging concern, 2013-14. River flows northwest.

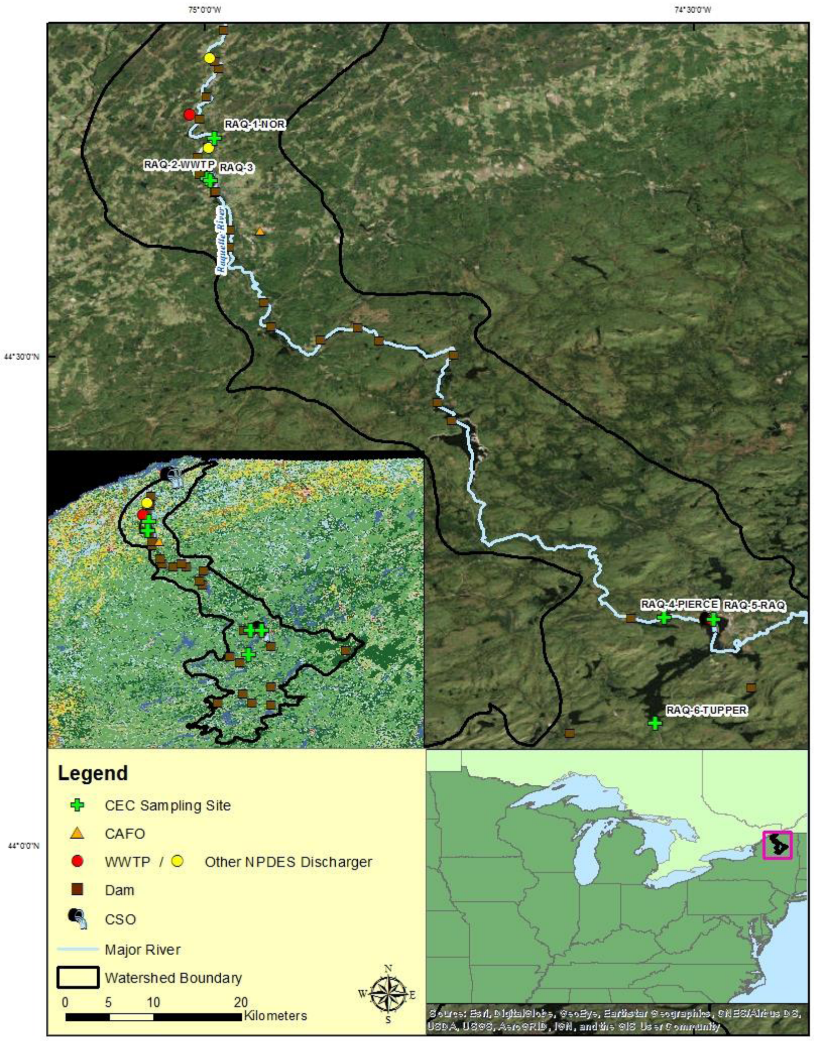

Fig K. Map of Raquette River sites sampled for contaminants of emerging concern, 2013-14. River flows northwest.
