# Supplementary material for: Contaminants of emerging concern in tributaries to the Laurentian Great Lakes: I. Patterns of occurrence
Source: PLoS One. 2017 Sep 27;12(9):e0182868. doi: 10.1371/journal.pone.0182868 (PMC5617142; doi:10.1371/journal.pone.0182868)
Supplement: S3 File — Sterols excluded because they comprised a relatively large percentage of total concentrations. The difference between 100 and total percent shown in graphs represents sterols. (PDF) [file pone.0182868.s008.pdf]

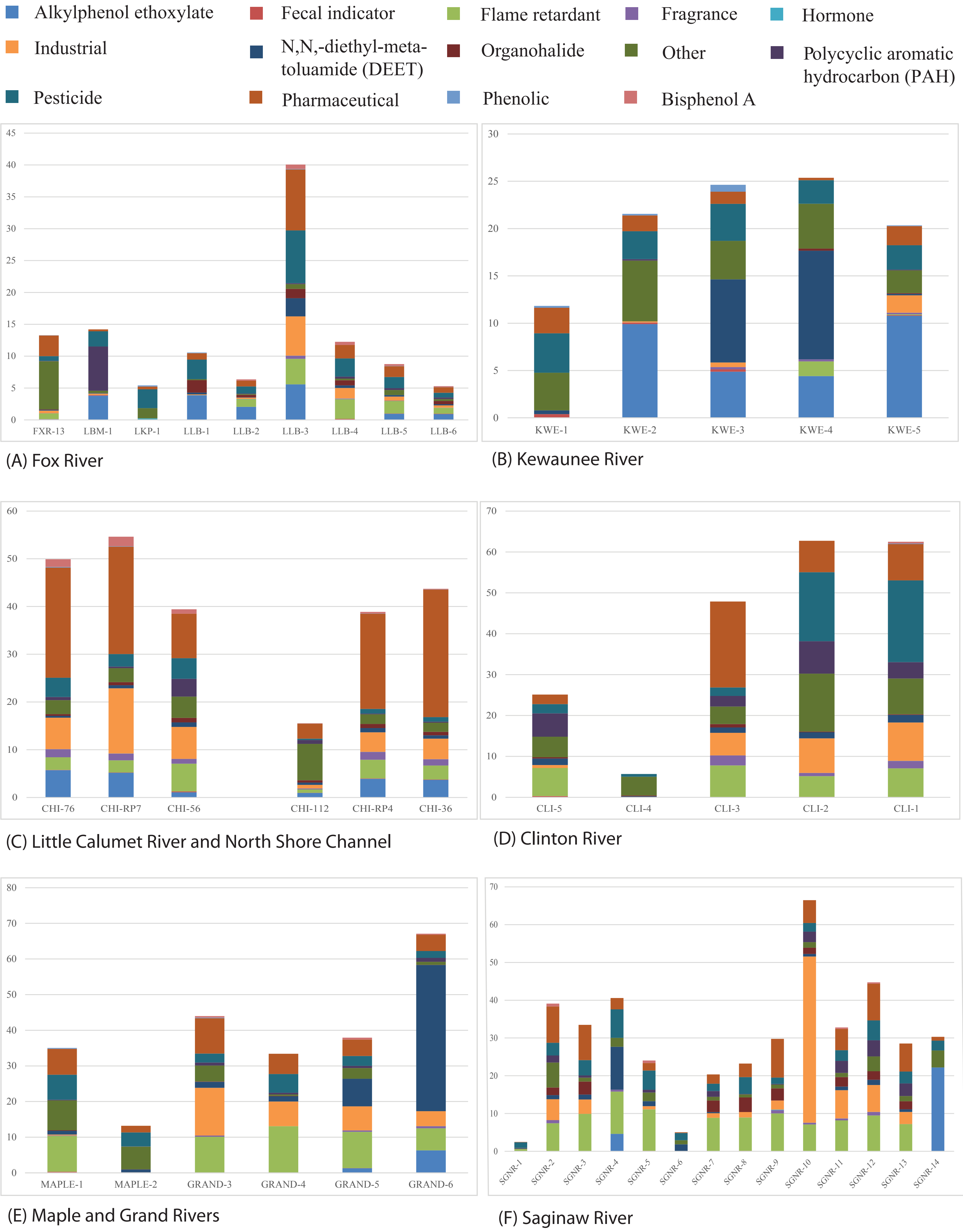
Alkylphenol ethoxylate
Fecal indicator
Flame retardant
Fragrance
Hormone
Industrial
N,N,-diethyl-meta-toluamide (DEET)
Organohalide
Other
Polycyclic aromatic hydrocarbon (PAH)
Pesticide
Pharmaceutical
Phenolic
Bisphenol A
FXR-13 LBM-1 LKP-1 LLB-1 LLB-2 LLB-3 LLB-4 LLB-5 LLB-6
(A) Fox River
KWE-1 KWE-2 KWE-3 KWE-4 KWE-5
(B) Kewaunee River
CHI-76 CHI-RP7 CHI-56 CHI-112 CHI-RP4 CHI-36
(C) Little Calumet River and North Shore Channel
CLI-5 CLI-4 CLI-3 CLI-2 CLI-1
(D) Clinton River
MAPLE-1 MAPLE-2 GRAND-3 GRAND-4 GRAND-5 GRAND-6
(E) Maple and Grand Rivers
SGNR-1 SGNR-2 SGNR-3 SGNR-4 SGNR-5 SGNR-6 SGNR-7 SGNR-8 SGNR-9 SGNR-10 SGNR-11 SGNR-12 SGNR-13 SGNR-14
(F) Saginaw River

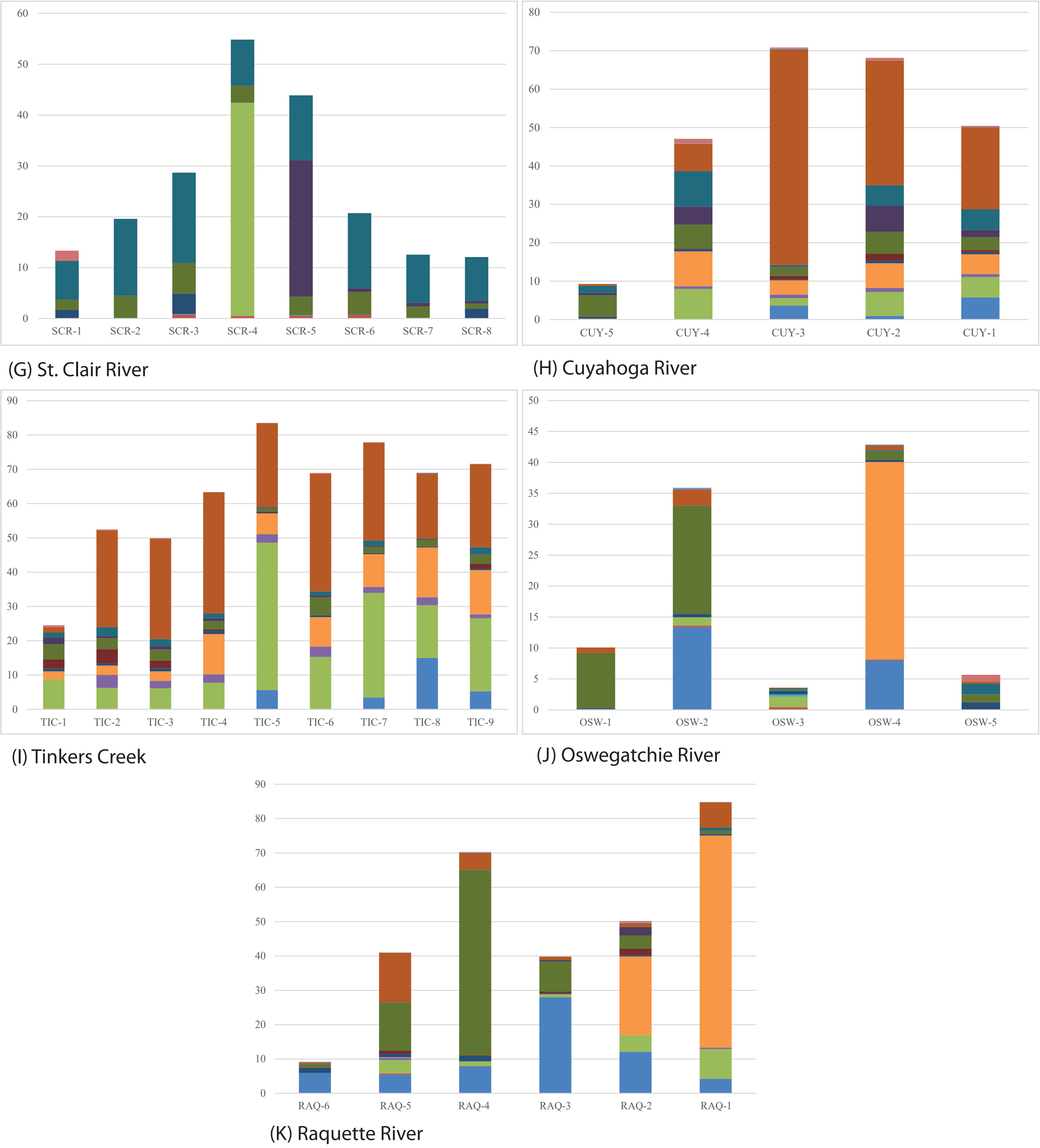

S12 Fig. Relative concentrations (based on maximum concentrations reported in S3 Table) of chemical classes in water samples from U.S. tributaries to the Great Lakes. Sterols excluded becasue they comprised a relatively large percentage of total concentrations. The difference between 100 and total percent shown in graphs represents sterols.
