# Supplementary material for: Contaminants of emerging concern in tributaries to the Laurentian Great Lakes: I. Patterns of occurrence
Source: PLoS One. 2017 Sep 27;12(9):e0182868. doi: 10.1371/journal.pone.0182868 (PMC5617142; doi:10.1371/journal.pone.0182868)
Supplement: S4 File — Sterols excluded because they comprised a relatively large percentage of total concentrations. The difference between 100 and total percent shown in graphs represents sterols. (PDF) [file pone.0182868.s009.pdf]

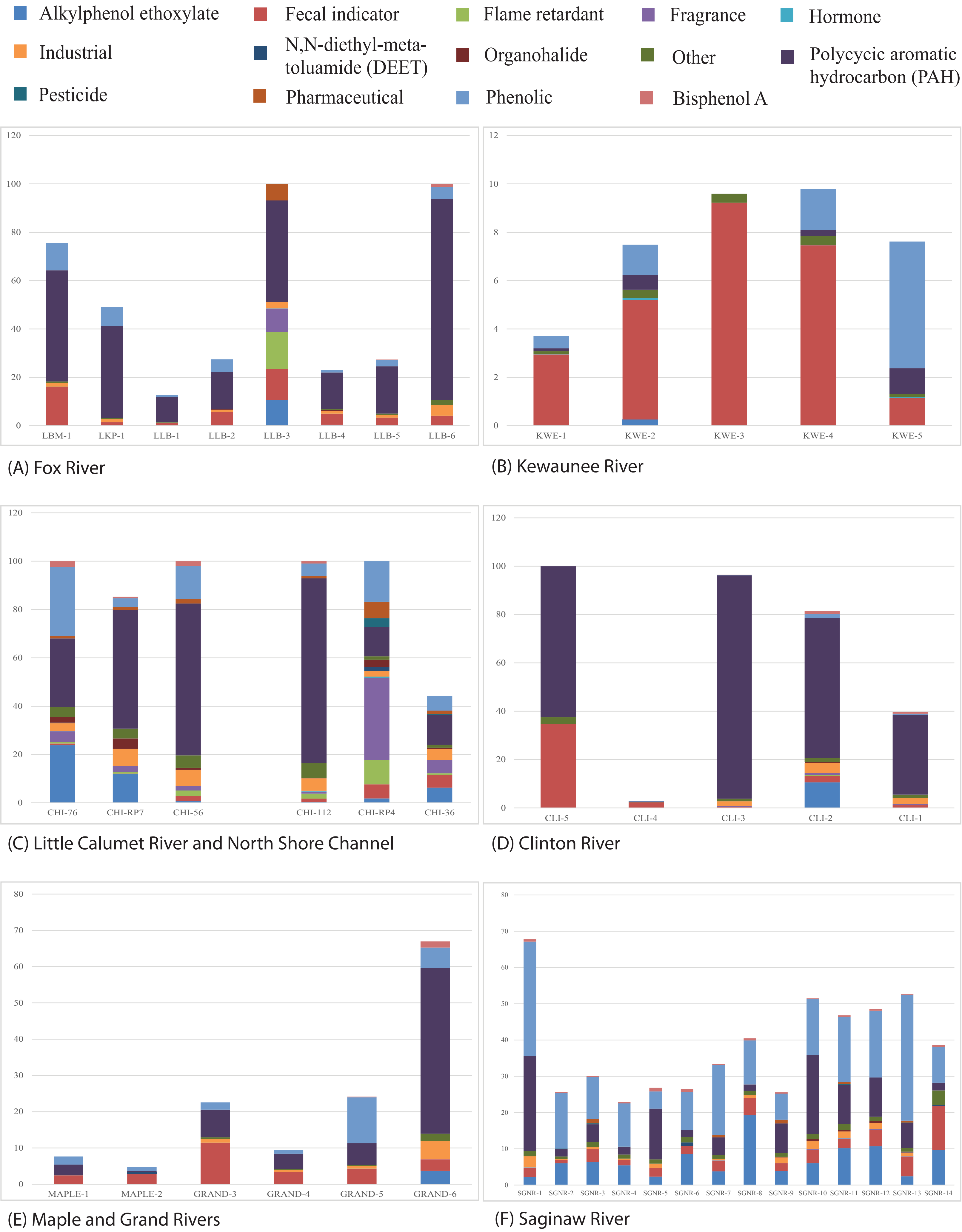
Alkylphenol ethoxylate
Fecal indicator
Flame retardant
Fragrance
Hormone
Industrial
N,N-diethyl-meta-toluamide (DEET)
Organohalide
Other
Polycycic aromatic hydrocarbon (PAH)
Pesticide
Pharmaceutical
Phenolic
Bisphenol A
0
20
40
60
80
100
120
LBM-1
LKP-1
LLB-1
LLB-2
LLB-3
LLB-4
LLB-5
LLB-6
(A) Fox River
0
2
4
6
8
10
12
KWE-1
KWE-2
KWE-3
KWE-4
KWE-5
(B) Kewaunee River
0
20
40
60
80
100
120
CHI-76
CHI-RP7
CHI-56
CHI-112
CHI-RP4
CHI-36
(C) Little Calumet River and North Shore Channel
0
20
40
60
80
100
120
CLI-5
CLI-4
CLI-3
CLI-2
CLI-1
(D) Clinton River
0
10
20
30
40
50
60
70
80
MAPLE-1
MAPLE-2
GRAND-3
GRAND-4
GRAND-5
GRAND-6
(E) Maple and Grand Rivers
0
10
20
30
40
50
60
70
80
SGNR-1
SGNR-2
SGNR-3
SGNR-4
SGNR-5
SGNR-6
SGNR-7
SGNR-8
SGNR-9
SGNR-10
SGNR-11
SGNR-12
SGNR-13
SGNR-14
(F) Saginaw River

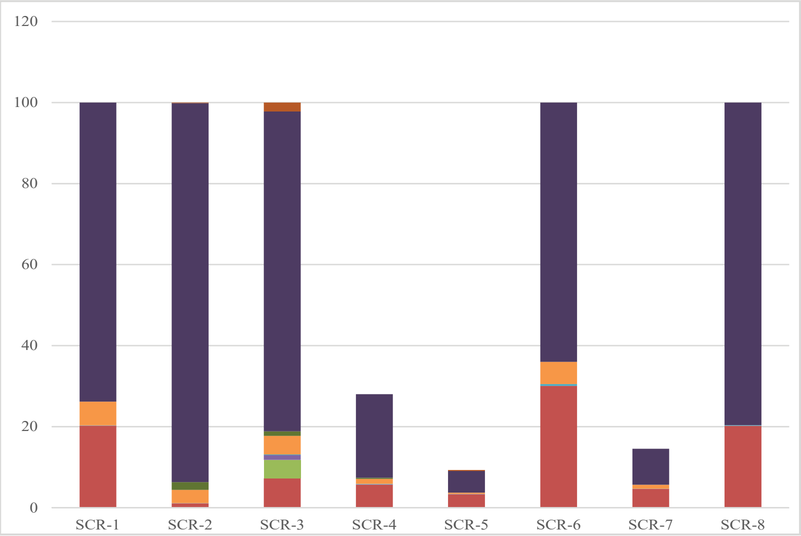

(G) St. Clair River

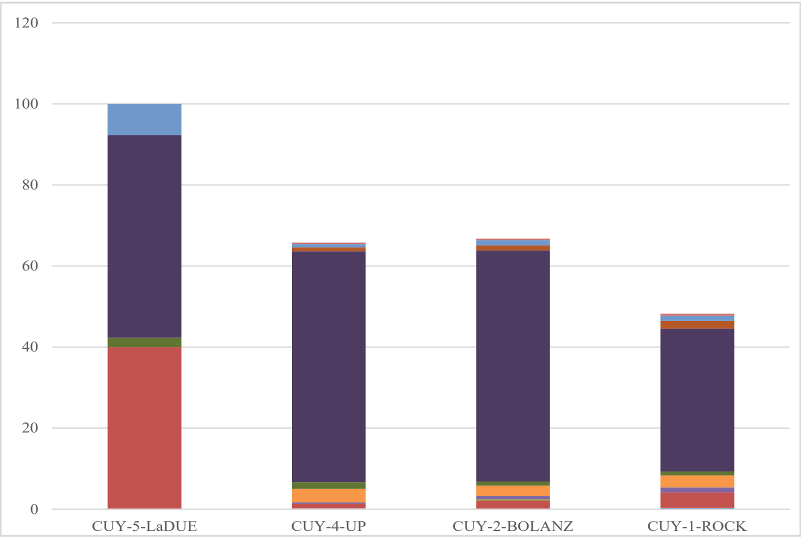

(H) Cyahoga River

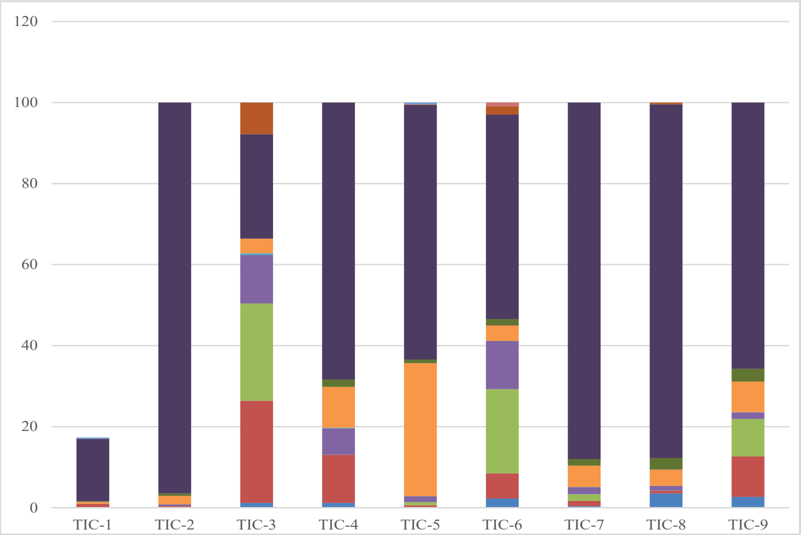

(I) Tinkers Creek

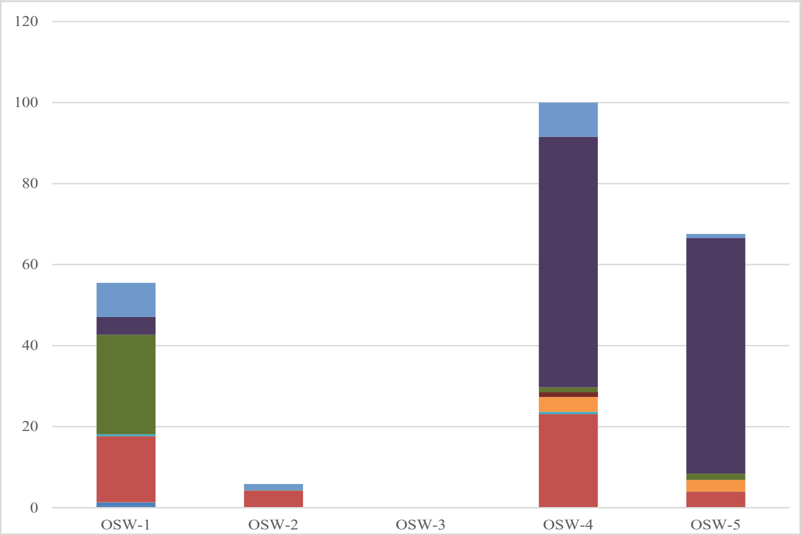

(J) Oswegatchie River

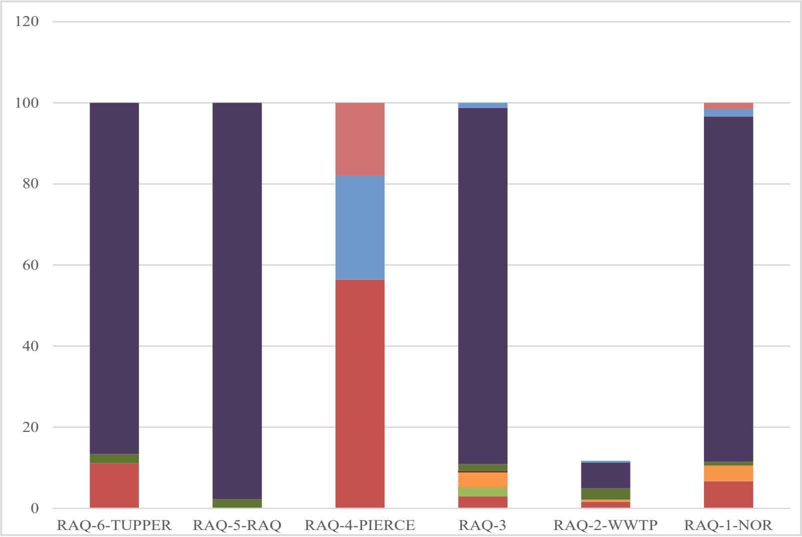

(K) Raquette River

S13 Fig. Relative concentrations (based on maximum concentrations reported in S3 Table) of chemical classes in sediment samples from U.S. tributaries to the Great Lakes. Sterols excluded because they comprised a relatively large percentage of total concentrations. The difference between 100 and total percent shown in graphs represents sterols.
